# Thermosensitive hydrogel with emodin-loaded triple-targeted nanoparticles for a rectal drug delivery system in the treatment of chronic non-bacterial prostatitis

**DOI:** 10.1186/s12951-023-02282-7

**Published:** 2024-01-18

**Authors:** Yan Ye, Wenzhen Zhong, Ruifeng Luo, Hongzhi Wen, Ziyang Ma, Shanshan Qi, Xiaoqin Han, Wenbiao Nie, Degui Chang, Runchun Xu, Naijing Ye, Fei Gao, Peihai Zhang

**Affiliations:** 1 TCM Regulating Metabolic Diseases Key Laboratory of Sichuan Province, Hospital of Chengdu University of Traditional Chinese Medicine, Chengdu, 610072 China; 2 State Key Laboratory of Southwestern Chinese Medicine Resources, Pharmacy School, Chengdu University of Traditional Chinese Medicine, Chengdu, 611130 China

**Keywords:** Chronic non-bacterial prostatitis, Emodin, Thermosensitive hydrogel, Rectal administration

## Abstract

**Background:**

The complex etiology and pathogenesis underlying Chronic Non-Bacterial Prostatitis (CNP), coupled with the existence of a Blood Prostate Barrier (BPB), contribute to a lack of specificity and poor penetration of most drugs. Emodin (EMO), a potential natural compound for CNP treatment, exhibits commendable anti-inflammatory, anti-oxidant, and anti-fibrosis properties but suffers from the same problems as other drugs.

**Methods:**

By exploiting the recognition properties of lactoferrin (LF) receptors that target intestinal epithelial cells (NCM-460) and prostate epithelial cells (RWPE-1), a pathway is established for the transrectal absorption of EMO to effectively reach the prostate. Additionally, hyaluronic acid (HA) is employed, recognizing CD44 receptors which target macrophages within the inflamed prostate. This interaction facilitates the intraprostatic delivery of EMO, leading to its pronounced anti-inflammatory effects. A thermosensitive hydrogel (CS-Gel) prepared from chitosan (CS) and β-glycerophosphate disodium salt (β-GP) was used for rectal drug delivery with strong adhesion to achieve effective drug retention and sustained slow release. Thus, we developed a triple-targeted nanoparticle (NPs)/thermosensitive hydrogel (Gel) rectal drug delivery system. In this process, LF, with its positive charge, was utilized to load EMO through dialysis, producing LF@EMO-NPs. Subsequently, HA was employed to encapsulate EMO-loaded LF nanoparticles via electrostatic adsorption, yielding HA/LF@EMO-NPs. Finally, HA/LF@EMO-NPs lyophilized powder was added to CS-Gel (HA/LF@EMO-NPs Gel).

**Results:**

Cellular assays indicated that NCM-460 and RWPE-1 cells showed high uptake of both LF@EMO-NPs and HA/LF@EMO-NPs, while Raw 264.7 cells exhibited substantial uptake of HA/LF@EMO-NPs. For LPS-induced Raw 264.7 cells, HA/LF@EMO-NPs can reduce the inflammatory responses by modulating TLR4/NF-κB signaling pathways. Tissue imaging corroborated the capacity of HA/LF-modified formulations to breach the BPB, accumulating within the gland's lumen. Animal experiments showed that rectal administration of HA/LF@EMO-NPs Gel significantly reduced inflammatory cytokine expression, oxidative stress levels and fibrosis in the CNP rats, in addition to exerting anti-inflammatory effects by inhibiting the NF-κB signaling pathway without obvious toxicity.

**Conclusion:**

This triple-targeted NPs/Gel rectal delivery system with slow-release anti-inflammatory, anti-oxidant, and anti-fibrosis properties shows great potential for the effective treatment of CNP.

**Graphical Abstract:**

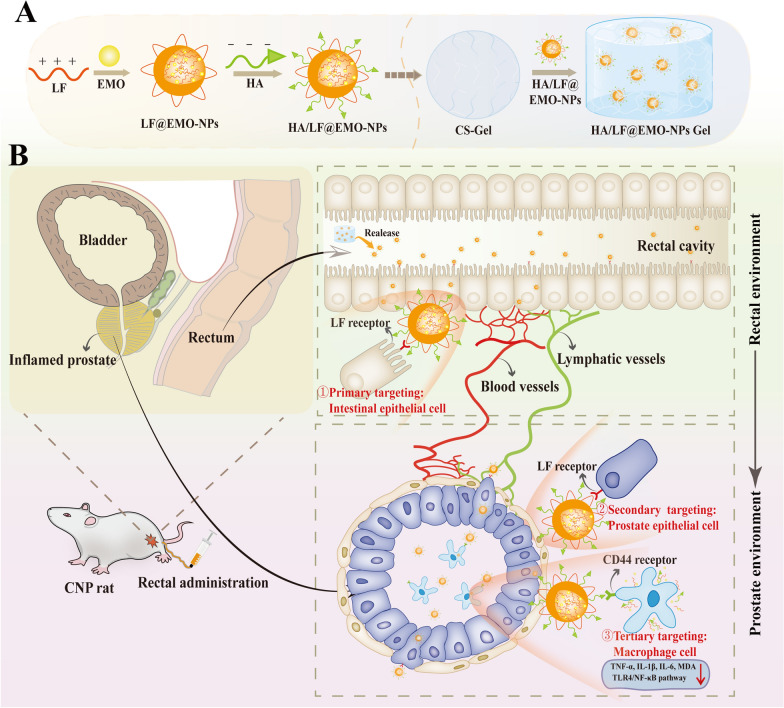

**Supplementary Information:**

The online version contains supplementary material available at 10.1186/s12951-023-02282-7.

## Introduction

Chronic prostatitis (CP) is a syndrome characterized by pain, urinary and sexual dysfunction, or varying degrees of psychosocial impairment [1]. This condition detrimentally affects the quality of life for up to 50% of men at some point in their lives [2, 3]. Notably, inflammation within the prostate gland plays a pivotal role in driving prostate cancer, primarily by inducing DNA damage and mutagenesis in the prostate epithelium. Remarkably, approximately 18% of prostatitis patients will eventually develop prostate cancer, which stands as the second leading cause of cancer-related death in males [4–6].

According to the National Institutes of Health (NIH) classification, more than 90% of CP cases are classified as chronic non-bacterial prostatitis (CNP) [7]. In addition, CNP patients comprise a substantial portion, accounting for 25% of global urologic outpatient visits [2]. The etiology and pathogenesis of CNP are complex and unclear, including infection, autoimmunity, endocrine imbalance, neuroplasticity and psychosocial conditions. However, the inflammatory response stands as the central mechanism driving the progression of the disease [8, 9]. Currently, common treatments for CNP include antibiotics, α-blockers, nonsteroidal anti-inflammatory drugs, etc. [10]. Yet, the distinctive physiological structure of the prostate gland poses challenges, including the presence of the blood-prostate barrier (BPB), hindering most drugs' ability to reach the prostate tissue and exert therapeutic effects. Additional factors such as low lipid solubility, low dissociation constants, and potential side effects further contribute to limited efficacy [11, 12]. Therefore, there is a need to find a biologically active drug that can easily penetrate the prostate epithelium to reach the prostate lumen and exert anti-inflammatory effects to treat CNP.

Phytotherapy stands out for its safety, efficacy, and minimal side effects, making it a reliable avenue for identifying CNP treatment agents [13]. Emodin (6-methyl-1,3,8-trihydroxyanthraquinone, EMO) is a hydrophobic anthraquinone compound extracted from *Rheum palmatum* and *Polygonum multiflorum* with good anti-inflammatory, anti-oxidant and and anti-fibrosis bioactivities [14]. Our research team found that EMO can exert anti-inflammatory effects through modulation of the Toll-like receptor 4/nuclear factor kappa-B (TLR4/NF-κB) signaling pathway for the treatment of ulcerative colitis [15]. In addition, EMO is also widely used in various inflammatory diseases, such as osteomyelitis, pneumonia and nephritis [16, 17]. Building on these findings, our previous research delved into the promising anti-inflammatory effects of EMO in the context of CNP. Nevertheless, owing to EMO's low water solubility and limited oral bioavailability, it faces challenges in traversing the BPB bottleneck, curtailing its clinical utilization [18].

Nanodrug delivery systems may be an effective way to break through BPB for the treatment of CNP [19, 20]. Prior studies have explored this avenue; however, many of these studies focused on invasive intravenous drug delivery. For the treatment of prostatitis, the safer and more suitable alternative lies in gentle rectal drug delivery [21, 22]. On the one hand, the prostate gland is adjacent to the rectum with rich venous plexus and lymphatic network traffic; on the other hand, rectal administration can avoid the first-pass effect and act directly on the prostate gland, increasing the local drug concentration and promoting the dissipation of inflammation in the glandular tissue [23, 24]. To ensure effective drug retention in the rectal area and perfect delivery to the prostate, we selected two materials, lactoferrin (LF) and thermosensitive hydrogel. Our thorough investigation into the intestinal microenvironment unveiled LF's specific affinity for lactoferrin receptors (LFRs), prominently expressed in intestinal epithelial cells, and the selection of LF as a carrier can facilitate intestinal absorption of drugs [15, 25, 26]. The thermosensitive hydrogel (CS-Gel), constituted from chitosan (CS) and β-glycerophosphate disodium salt (β-GP), exhibits biodegradability and malleability. The transition from solution to hydrogel occurs at rectal temperatures, ensuring prolonged drug retention in the rectum and enhancing drug uptake by intestinal epithelial cells [27, 28]. In addition, we found that LFR is also highly expressed in inflamed prostate epithelial cells and that absorbed drugs in the rectum are targeted to prostate epithelial cells via LF recognition, thus allowing drug delivery to the prostate [29, 30]. To ensure that the drug reaches the site of inflammation, hyaluronic acid (HA) was chosen for modification because of the presence of overexpressed CD44 receptors on macrophages at the site of prostatitis lesions. HA's ability to selectively recognize CD44 receptors makes it a promising candidate for targeting inflammation sites and treating CNP [31–33]. Based on this, drug absorption through the intestine into the diseased prostate and target recognition of the lesion is an intelligent choice.

**Scheme 1:**
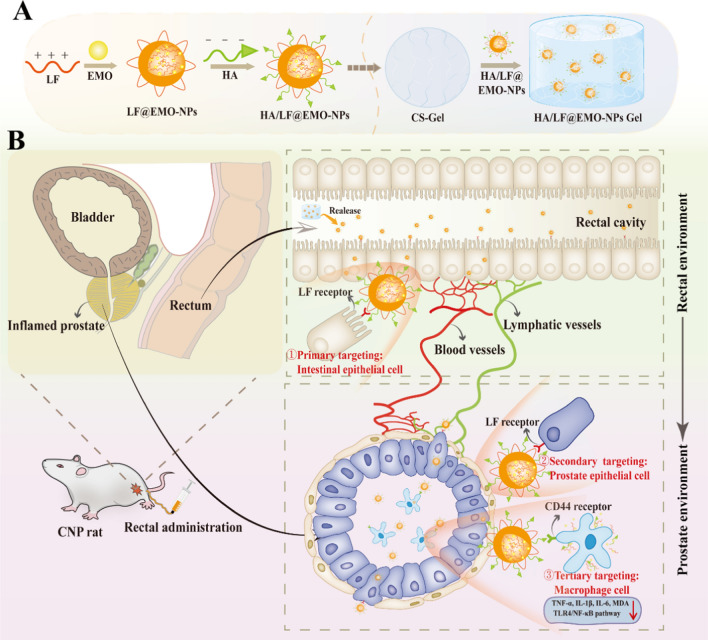
Schematic illustration of HA/LF@EMO-NPs Gel for CNP therapy. **A** Scheme showing the preparations of HA/LF@EMO-NPs Gel. **B** HA/LF@EMO-NPs Gel is administered rectally and hitchhikes sequentially on intestinal epithelial cells, prostate epithelial cells and macrophages to be delivered to the ectopic lesion, ultimately relieving CNP

In our study, positively charged LF was loaded with EMO through dialysis, forming LF@EMO-NPs. These were then combined with negatively charged HA through electrostatic interactions, generating HA/LF@EMO-NPs. Finally, HA/LF@EMO-NP lyophilized powder was incorporated into CS-Gel, creating a triple-targeted thermosensitive hydrogel (HA/LF@EMO-NPs Gel) designed for rectal drug delivery. We characterized the physicochemical properties of the NPs/Gel and evaluated the contribution of the materials in the system to rectal drug delivery, including targeting of intestinal epithelial cells, rectal retention, and targeting of inflammation sites. Crucially, we observed, for the first time, the therapeutic effects of nano-sized EMO on CNPs. We validated its anti-inflammatory properties through the TLR4/NF-κB classical inflammatory signaling pathway, highlighting EMO's antioxidant and anti-fibroproliferative potential for CNPs (Scheme 1).

## Materials and methods

### Materials

EMO was purchased from Dalian Meilun Biotech Co., Ltd. (Dalian, China). LF was obtained from Shanghai Yuanye Bio-Technology Co., Ltd. (Shanghai, China), and HA was provided by Bloomage Biotechnology Co., Ltd. (Shandong, China). CS and β-GP were purchased from Shanghai Aladdin Biochemical Technology Co., Ltd. (Shanghai, China). Dimethyl sulfoxide (DMSO) was purchased from J&K Scientific (Beijing, China). Coumarin-6 (C_6_) was purchased from Aladdin Reagent Company (Shanghai, China), and an antifade mounting medium with DAPI was obtained from Beyotime Biotechnology Co., Ltd. (Shanghai, China). The tumor necrosis factor-alpha (TNF-α) kit, interleukin-1β (IL-1β) kit, and interleukin-6 (IL-6) kit were purchased from MultiScience (Lianke) Biotech Co., Ltd. (Hangzhou, China). The malondialdehyde (MDA) kit was purchased from ElabScience Biotech Co., Ltd. (Wuhan, China). Anti-myeloid differentiation factor 88 (MyD88) and anti-TLR4 antibodies were supplied by Servicebio Technology Co., Ltd. (Wuhan, China), and anti-NF-κB p65 was provided by ImmunoWay Biotechnology Company (St. Plano, TX, USA). All chemicals used in the study were not further purified.

### Preparation of LF@EMO-NPs and HA/LF@EMO-NPs

LF@EMO-NPs were prepared using a slightly modified dialysis [15]. In brief, 100 mg LF was dissolved in 40 mL ultrapure water, along with the dissolution of 10 mg EMO in 2 mL DMSO. Then 2 ml of EMO solution was dripped into 40 ml of LF solution. To improve the solubility of EMO, the above solutions were sonicated for 5 min using a probe sonicator (Sonicator XL; Misonix, Melville, NY, USA), kept on a magnetic stirrer for 30 min to ensure uniform distribution and optimum size reduction, and dialyzed in ultrapure water with a dialysis bag (MWCO 1000, Millipore, USA) for 8 h. LF@EMO-NPs were passed through a syringe filter (0.22 μm) to remove impurities and free drug.

HA/LF@EMO-NPs were prepared by electrostatic adsorption [26]. Briefly, 4 mg of HA was dissolved in 2 mL of ultrapure water, followed by dropwise addition of HA solution to the LF@EMO-NPs solution after filtration. After stirring for 30 min and filtering through a syringe filter (0.22 μm), HA/LF@EMO-NPs were lyophilized until further use. All the above steps were performed under light-proof conditions.

### Characterization of LF@EMO-NPs and HA/LF@EMO-NPs

The average hydrodynamic particle size, polydispersity index (PDI), and zeta potential of NPs were measured by dynamic light scattering (DLS) using a Particle Analyzer Litesizer 500 (Anton Paar, Graz, Austria). Average values were obtained from three replicate experiments.

The morphology of the NPs was analyzed by transmission electron microscopy (TEM, JEM 1200X; JEOL, Japan) after staining with 2% phosphotungstic acid. Moreover, the X-ray powder diffraction (XRD, Rigaku Ultma IV, Japan) spectra of free EMO, free LF, free HA, blank NPs, HA/LF@EMO-NPs, and mixed free EMO, LF and HA were analyzed by scanning from 5 to 90° at 40 kV and 40 mA.

To determine the encapsulation efficiency (EE) and loading efficiency (LE) of EMO, we used methanol to disrupt the structure of NPs and dissolve EMO and then determined them by high-performance liquid chromatography (HPLC, LC-45202-46, Shimadzu, Japan). Methanol/0.1% phosphoric acid (85/15, v/v) was chosen as the mobile phase. The flow rate was set at 1 mL/min, and the detection wavelength was 254 nm. EE and LE were calculated using the following equations:

1 $${\text{EE }}\left( \% \right) \, = \, \left( {\text{Actual EMO loading}} \right)/\left( {\text{Theoretical EMO loading}} \right) \, \times {1}00\%$$

2 $${\text{LE }}\left( \% \right) \, = \, \left( {\text{Weight of EMO in NPs}} \right)/\left( {\text{Weight of NPs}} \right) \, \times {1}00\%$$

### Preparation of HA/LF@EMO-NPs gel

To increase the retention of NPs in the rat rectum, in this study, they were encapsulated in a hydrogel composed of CS and β-GP to obtain HA/LF@EMO-NPs Gel [27, 34, 35]. We investigated different mass ratios of CS and β-GP solutions to prepare the optimal thermosensitive rectal hydrogel. We dissolved CS in 0.1 M dilute hydrochloric acid for the preparation of a homogeneous CS solution with a concentration of 2%. Then, CS/β-GP solutions with mass ratios of 1:1.5, 1:2, 1:2.5, 1:3, 1:3.5, 1:4, and 1:4.5 were prepared by dissolving β-GP powder in ultrapure water at a fixed volume. Finally, HA/LF@EMO-NPs lyophilized powder was added to the CS/β-GP solution under ice bath conditions with gentle stirring to disperse the NPs well. This mixture could spontaneously form HA/LF@EMO-NPs Gel at 37 °C.

### Characterization of HA/LF@EMO-NPs gel

The gelation time of the Gel was determined using the inversion method. Various combinations of hydrogels were thoroughly mixed and subsequently placed in a water bath at a constant temperature of 37 °C. The samples were inverted every 20 s during this process. The gelation time, defined as the point at which the sample ceased to flow, was meticulously recorded. To ensure accuracy, three parallel samples were analyzed, and the average values were calculated from these measurements. The pH of the gel was measured posterior to gelation using a calibrated pH meter (Hanna, model 211, Romania). The readings were recorded in triplicate, and the average reading was calculated.

The mechanical strength of Gel was measured by a homemade heavy load device. The procedure involved excising two approximately 1.0 cm long segments of rat rectal tissues, longitudinally cutting them, and placing them on glass slides with the mucosa facing outward. An appropriate amount of the final preparation (in solution state) was added to the inner wall of the lower rectal tissue in a pre-ice bath, and the solution was placed between the two rectal tissues by pressing on the slides for 3 min to remove air bubbles and bring the rectum into full contact with the solution. After being placed in a 37 °C incubator, the solution-hydrogel transition was completed. Subsequent to this, the assembly was placed within a 37 °C incubator to complete the transition of the solution to the hydrogel state. Upon reaching complete solidification, the assembly was oriented vertically on an iron stand, as illustrated in Additional file 1: Fig. S1. Briefly, a sealed bag was mounted on the lower glass slide, ultrapure water was added dropwise until the glass slide dropped due to reaching the loading limit, and the weight was recorded. Each sample was measured three times in parallel, and the average value was obtained.

We used a scanning electron microscope (SEM; Gemini 300; ZEISS, Germany) to observe the morphology of Gel. Before SEM, dried HA/LF@EMO-NPs Gel samples were sputtered with gold under a high vacuum.

### In vitro release studies

The release profiles of EMO from various formulations were investigated using phosphate-buffered saline (PBS; pH 7.4) containing 0.5% Tween-80 as the release medium. In this study, dialysis bags with a molecular weight cutoff of 3000 (Millipore, USA) were utilized. These bags were filled with 3 mL of free EMO, HA/LF@EMO-NPs, free EMO Gel, and HA/LF@EMO-NPs Gel, respectively. The filled bags were then submerged in 30 mL of the release medium and maintained at a temperature of 37 °C with continuous agitation at 100 rpm. At designated time intervals (1, 2, 4, 8, 12, 18, 24, and 36 h), 1 mL of sample was removed, 1 mL of fresh media was added, and the concentration of EMO in the sample taken was determined by HPLC. By calculating the cumulative percentage of EMO released over time, we constructed corresponding graphs illustrating the temporal variations in EMO release. Notably, to evaluate that NPs, rather than EMO or partially modified NPs, are released from the Gel, we have collected the dialysis solution after HA/LF@EMO-NPs Gel release, then measured the particle size by DLS, and observed the morphology by TEM.

### Cell culture

NCM-460 cells were cultured in RPMI-1640 containing 10% fetal bovine serum (FBS) supplemented with 1% streptomycin and penicillin, respectively. RAW 264.7 and Caco-2 cells were cultured in DMEM containing 10% fetal bovine serum (FBS) supplemented with 1% streptomycin and penicillin, respectively. All reagents above were provided by Thermo Fisher Scientific. RWPE-1 cells were cultured in KM (ScienCell Research Laboratories, Inc.) with 1% keratinocyte growth supplement (KGS, Cat. #2152) and 1% penicillin/streptomycin solution (P/S, Cat. #0503). The drug was diluted with incomplete medium.

### In vitro cellular uptake

Cellular uptake is the major indicator for in vitro evaluation of nanodrug performance. The uptake of NPs by cells was measured qualitatively by confocal laser scanning microscopy (CLSM, TCS SP8 SR, Leica, Weztlar, Germany) and quantitatively by flow cytometry (FCM, NovoCyte, ACEA, San Diego, CA, USA). NCM-460, RAW 264.7 and RWPE-1 cells were used as cellular models to evaluate the internalization of different NPs. The nuclei were labeled with blue fluorescent DAPI, and C_6_-NPs were prepared using the green fluorescent probe C_6_ instead of EMO, which showed no significant fluorescence.

The three cell types, namely NCM-460, RWPE-1, and RAW 264.7 cells, were cultured in close proximity within confocal culture dishes until they became adherent. They were then co-incubated with free C_6_, LF@C_6_-NPs and HA/LF@C_6_-NPs (100 ng/mL C_6_) for 4 h to compare the difference in uptake of different C_6_-loaded formulations. To investigate whether LF and HA have targeting effects, we preincubated NCM-460 and RWPE-1 cells with LF and RAW 264.7 cells with HA for 2 h. Then, they were washed with PBS before incubation with LF@C_6_-NPs and HA/LF@C_6_-NPs (100 ng/mL C_6_) for 4 h. Finally, the cells were thoroughly washed with refrigerated PBS to eliminate excess reagents, followed by fixation with 4% paraformaldehyde for 10 min and staining with DAPI solution for subsequent CLSM imaging analysis.

Quantitative analysis of cellular uptake. The treatment of cells was consistent with CLSM. We incubated cells with different concentrations (0, 6.25, 12.5, 25, 50, 100 ng/mL) of HA/LF@C_6_-NPs at 37 °C for 4 h to study the concentration dependence of cells for NPs and incubated with uniform concentrations of HA/LF@C_6_-NPs (100 ng/mL C_6_) at different time points (0, 0.25, 0.5, 1, 2, 4 h) to study the time dependence of cells for NPs. Subsequent to these treatments, cells were rinsed with cold PBS, centrifuged at 4 °C, resuspended in 1 mL of sterile PBS, and transferred to flow tubes at a density of 2 × 10^5^ cells per tube in preparation for flow cytometry analysis. Notably, all experiments integrated untreated cells as control references.

### In vitro NPs transport studies

Given that in vivo experiments are difficult to represent the process, therefore we validated it by establishing an in vitro model. Relevant studies have shown that the intestinal epithelial cell monolayer model established by Caco-2 cells has been widely used for drug uptake transporter studies [36, 37]. Therefore, we inoculated Caco-2 cells in the upper chamber of Transwell plates and cultured them for 21 d to construct the intestinal epithelial monolayer cell model, and when the resistance was > 500 Ω/cm^2^, we inoculated RWPE-1 cells in the lower chamber. Then they were divided into 3 groups for subsequent experiments: Free C_6_, LF@C_6_-NPs, and LF-saturated group. The upper chamber was administered in the same way as method 2.8, and CLSM was applied to detect the fluorescence intensity of PWPE-1 in the lower chamber of each group.

#### In vitro anti-inflammatory efficacies and mechanisms of NPs

To examine in vitro anti-inflammatory efficacies and mechanisms of NPs. LPS-induced RAW264.7 cells were used as the cell model [38]. Briefly, RAW 264.7 cells in stable growth state were taken and inoculated in 12-well plates at a density of 2.0 × 10^5^/mL per well, and divided into 5 groups for the experiment: Control group, LPS group, LPS + drug-containing group (Free EMO, LF@EMO-NPs, HA/LF@EMO-NPs). LPS 1 µg/mL was incubated alone for 12 h, then replaced with fresh medium or drug-containing medium (EMO 10 µM) to continue incubation for 12 h [39]. Cell-free supernatants were collected for pro-inflammatory cytokine assays using a ELISA kit according to the manufacturer's instructions. The anti-inflammatory effects (TLR4/MyD88/NF-κB p65) in LPS-induced RAW 264.7 cells were detected using Western blotting.

#### Targeting capability of gel

We investigated the accumulation of different formulations in prostate, rectal, and other organs (heart, liver, spleen, lung, and kidney) tissues using CLSM to confirm the potential targeting ability of the Gel. EMO has no significant fluorescence; therefore, C_6_ was used as a fluorescent probe to prepare free C_6_, LF@C_6_-NPs, HA/LF@C_6_-NPs and HA/LF@C_6_-NPs Gel. Briefly, CNP rats were administered the above formulations at a single dose 24 h after rectal administration. Prostate, rectal, and other organs (heart, liver, spleen, lung, and kidney) tissues were collected under light-proof conditions and embedded in Optimal Cutting Temperature compounds, sectioned, and stained with DAPI to detect C_6_ accumulation using CLSM. Furthermore, in order to evaluate the retention time of the thermosensitive gel in the rat rectum, we collected the residual gel in the rectum at different time points (1, 3, 6, 12, 24 h) after rectal administration of the HA/LF@EMO-NPs Gel, and the content of EMO in the gel was detected by HPLC (EMO Content = EMO (ug)/Gel (g)) [40, 41].

#### In vivo therapeutic evaluation

Sprague Dawley (SD) adult male rats (6–8 weeks, 220–240 g) were provided by SPF Biotechnology Co., Ltd. (Beijing, China) and stored at 22–25 °C and 50–60% humidity with a 12 h/12 h light/shadow cycle. The rats had free access to food and water. All animal procedures were conducted in accordance with the strict Guidelines for the Care and Use of Laboratory Animals of the Ministry of Science and Technology of China.

A carrageenan-induced CNP rat model was used for in vivo treatment evaluation. The rats were randomly classified into 8 groups: (1) normal group, (2) sham-operated (sham) group, (3) blank Gel group, (4) model group, (5) free EMO group, (6) LF@EMO-NPs group, (7) HA/LF@EMO-NPs group, and (8) HA/LF@EMO-NPs Gel group (n = 6 per group). For the model, blank Gel, free EMO, LF@EMO-NPs, HA/LF@EMO-NPs, and HA/LF@EMO-NPs groups, modeling was started after 1 week of acclimatization, and 100 μL of carrageenan was injected into each side of the prostate of each rat. The sham groups were each injected with 100 μL of saline. One week later, the CNP model was completed, and then 15 mg/kg of different EMO preparations were given to the administered group for 14 days; the same dose of saline was given to the normal, sham and model groups. After the final administration of the drug, the rats were sacrificed, and the prostate and major organs (heart, liver, spleen, lungs and kidneys) were dissected. The prostate gland was weighed individually to analyze the prostate index (PI, PI = weight of prostate (mg)/bodyweight (g). The prostate and other organs were then fixed in 4% formalin, embedded in paraffin, and sectioned (5 μm). After hematoxylin and eosin (H&E) staining and Masson staining, the sections were imaged under a microscope and the pathological scores were performed [42, 43]. Furthermore, the expression level of TNF-α, IL-6 in the serum of rats, and the expression level of TNF-α, IL-1β, IL-6, MDA in prostate tissues were measured by enzyme-linked immunoassay kits. In addition, we analyzed the expression of TLR4, MyD88, and NF-κB p65 using Western blotting. β-Actin antibody was used as an internal reference to determine protein equivalents in the sample. We analyzed the obtained chemiluminescence signals by ImageJ software.

#### Statistical analysis

All data were analyzed using GraphPad Prism 8.0 software and were measured at least three times independently. Differences between groups were analyzed using one-way ANOVA. A *p* value < 0.05 was considered statistically significant.

## Results and discussion

### Characterization of NPs and gel

#### NPs characterization

**Fig. 1:**
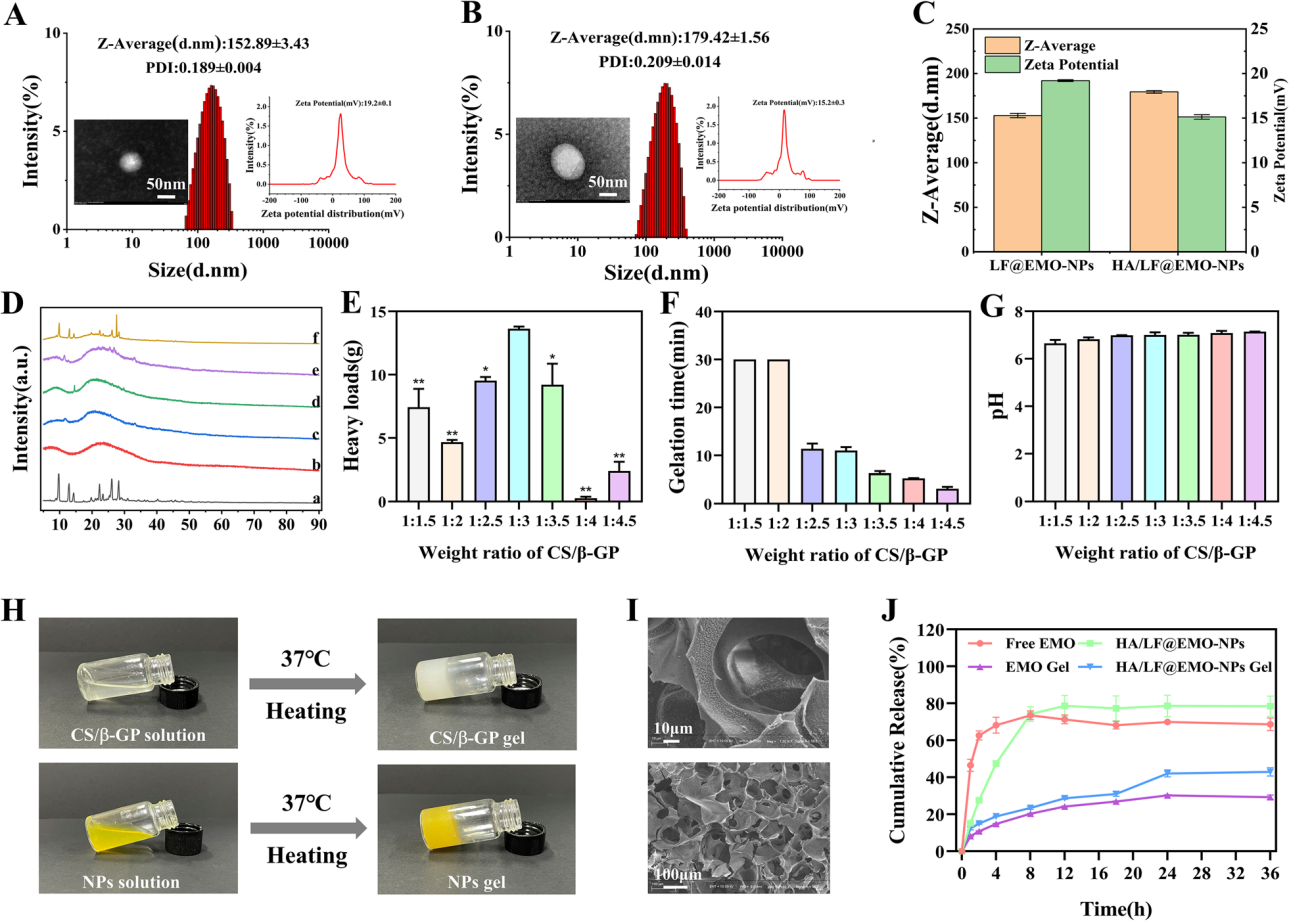
NPs and Gel characterizations. **A** Size, Zeta potential distributions and TEM of LF@EMO-NPs. **B** Size, Zeta potential distribution and TEM of HA/LF@EMO-NPs. **C** Comparison of size, Zeta potential distributions of LF@EMO-NPs and HA/LF@EMO-NPs. **D** XRD spectra of different preparations. **a** Free EMO, (**b**) Free LF, (**c**) Free HA, (**d**) Blank NPs, (**e**) HA/LF@EMO-NPs, and (**f**) mixed free EMO, LF and HA. **E**–**G** Heavy loads, Gelation time, pH of Gel with different weight ratio of CS/β-GP (vs 1:3). **H** Inverted vial experiments demonstrate Gel formation in the presence and absence of HA/LF@EMO-NPs. **I** SEM image of HA/LF@EMO-NPs Gel. **J** In vitro release profiles of EMO from Free EMO, HA/LF@EMO-NPs, EMO Gel and HA/LF@EMO-NPs Gel in PBS (pH 7.4) containing 0.5% Tween-80 at 37 °C. Data are represented as the mean ± SEM (n = 3). *p < 0.05; **p < 0.01

We employed dialysis and electrostatic adsorption reactions, as detailed in the preceding methods, to formulate nanoparticles (NPs). The average hydrodynamic diameter of LF@EMO-NPs was assessed using Dynamic Light Scattering (DLS), as depicted in Fig. 1A, yielding a measurement of 152.89 ± 2.43 nm. This measurement exhibited a singular peak distribution, indicated by a Polydispersity Index (PDI) of 0.189 ± 0.004. Moreover, the NPs exhibited a zeta potential of 19.2 ± 0.1 mV, indicating a substantial positive charge. The Transmission Electron Microscopy (TEM) image, also shown in Fig. 1A, displayed spherical and uniform LF@EMO-NPs structures. The Encapsulation Efficiency (EE) and Loading Efficiency (LE) of LF@EMO-NPs were determined to be 90.74% ± 0.88% and 8.22% ± 0.05%, respectively.

Following the introduction of HA, the average hydrodynamic diameter of HA/LF@EMO-NPs increased to 179.42 ± 1.56 nm, accompanied by a solitary peak distribution (PDI = 0.209 ± 0.014). The zeta potential exhibited a value of 15.2 ± 0.3 mV, and TEM imaging demonstrated the maintenance of a spherical and uniform structure (Fig. 1B). The EE and LE were 85.92% ± 1.34% and 7.54% ± 0.12%, respectively, both of which were higher and could improve efficacy and reduce drug dosage. We observed that the particle size of the first layer of NPs increased and the zeta potential decreased after the addition of negatively charged HA (Fig. 1C), which still showed a single-peak distribution, suggesting that HA was successfully adsorbed into the LF layer through electrostatic interactions.

X-Ray Diffraction (XRD) analysis, as shown in Fig. 1D, indicated that the free EMO group exhibited sharp peaks consistent with crystalline properties. Contrarily, the combination of EMO with all carrier matrices retained numerous crystalline peaks, distinct from the absence of non-crystalline peaks in other groups. This observation leads to the inference that EMO was highly soluble within the NPs and existed in an amorphous state. The mixture of EMO and all carrier matrixes still had many crystalline peaks in contrast to the non-crystalline peaks in other groups. Therefore, it can be speculated that EMO is highly soluble in NPs and exists in an amorphous state.

#### Gel characterization

The gel was prepared using the cold method previously listed in the Methods section. In this study, we examined the ratio between CS and β-GP, with viscosity as the primary indicator and gel time and pH as secondary indicators, to determine the optimal gel composition. Upon evaluation, it was evident that a mass ratio of CS to β-GP at 1:3 (as illustrated in Fig. 1E, F, G) yielded the most favorable results. This ratio yielded the highest viscosity, indicated by a substantial heavy load of approximately 14 g, surpassing other ratios [44]; The gel formation time was roughly 10 min at this composition, a duration chosen to prevent causing undue stress to the rats due to a shorter timeframe, which could lead to rapid drug excretion. Simultaneously, the pH remained approximately 7.0, maintaining compatibility with the rectal environment [45]. Then, HA/LF@EMO-NPs lyophilized powder was added to CS-Gel, which was in solution at low temperature and transformed into a semisolid gel (HA/LF@EMO-NPs Gel) at rectal temperature (Fig. 1H). In addition, HA/LF@EMO-NPs Gel was freeze-dried, and the structure was observed as homogeneous pores by SEM (Fig. 1I), which contributed to the sustained slow release of the drug.

### In vitro release studies

In order to substantiate the sustained release capability of the Gel, an in vitro drug release simulation was conducted (as shown in Fig. 1J). Complete release of free EMO and HA/LF@EMO-NPs was achieved at around 12 h, resulting in cumulative release rates of 74% and 78%, respectively. In contrast, free EMO Gel and HA/LF@EMO-NPs Gel were released slowly due to their porous structure, with cumulative release rates of approximately 30% and 42% in 36 h. These outcomes underscored the effective control of sustained EMO release achievable through encapsulation within the CS-Gel. Furthermore, it was evident that the slower release of free EMO Gel compared to HA/LF@EMO-NPs Gel might be attributed to the limited solubility of EMO, leading to restricted permeation out of the dialysis bag. Additionally, the DLS of the dialysis solution of the Gel (as illustrated in Additional file 1: Fig. S2A) showed that the nanoparticle size of 187.54 ± 1.54 nm with a single peak distribution (PDI = 0.184 ± 0.016). The increase in the size of the NPs is considered to be the result of the Gel being a thermosensitive gel formed by the re-dispersion of nanoparticle’s lyophilized powders, which were degraded first as NPs in water, at the same time, a certain degree of nanoparticle reassembly was carried out. TEM of the dialysis solution of the Gel (as illustrated in Additional file 1: Fig. S2B) showed that the structure of nanoparticles was spherical with a few fragments around them, which was considered to be the degradation of CS, β-GP and other substances constituting the Gel. The above results suggested that the Gel mainly released the drug outward in the form of NPs. Therefore, CS/β-GP hydrogels are an ideal vehicle for drug release control, and hydrogels loaded with NPs have important potential applications in biomedical fields.

### Cellular uptake

Effective cellular uptake stands as a pivotal prerequisite for the successful delivery of EMO-loaded nanodrugs, a crucial aspect in the treatment of CNP. Our investigation began by scrutinizing the targeting capacity of the food-derived protein LF on epithelial cell lines. Effective uptake of LF@EMO-NPs by intestinal epithelial cells (NCM-460) with high expression of LFR facilitated the smooth absorption of the drug through the intestine, as prostate epithelial cells (RWPE-1) also had high expression of LFR, and the drug absorbed through the intestine could reach the prostate. The targeting effect of food-derived polysaccharide HA on macrophages (RAW 264.7) was then investigated. HA displayed a distinctive ability to specifically recognize the CD44 receptor on RAW 264.7 cells, establishing binding interactions. Since EMO does not have significant fluorescence, we used the fluorescent probe C_6_ instead of EMO for tracking.

#### Uptake of LF@EMO-NPs by LFR-positive NCM-460 and RWPE-1 cells

LFR is overexpressed on the membranes of NCM-460 and RWPE-1 cells [29, 46]. Therefore, in this study, we used the aforementioned cells to assess the uptake ability of LF@C_6_-NPs.

**Fig. 2:**
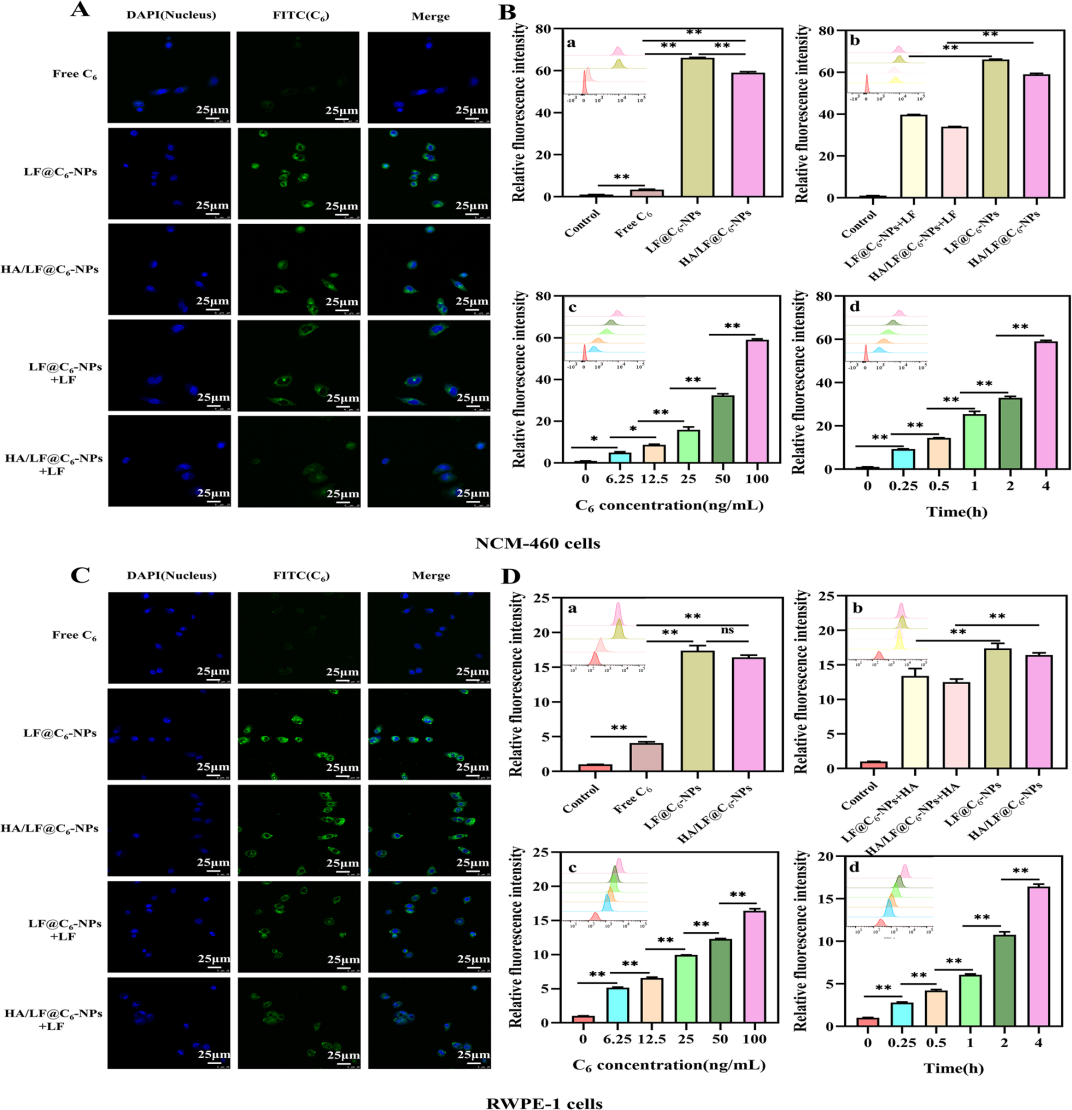
Cellular uptake evaluation of NCM-460 cells and RWPE-1 cells. **A** Qualitative analysis of uptake by NCM-460 cells incubated with Free C_6_, LF@C_6_-NPs, and HA/LF@C_6_-NPs with or without LF. **B** Quantitative analysis of uptake by NCM-460 cells. **a** incubated with Control, Free C_6_, LF@C_6_-NPs, and HA/LF@C_6_-NPs; **b** incubated with LF@C_6_-NPs and HA/LF@C_6_-NPs with or without LF; **c** Quantitative determination of HA/LF@C_6_-NPs uptake by NCM-460 cells after 4 h incubation at different concentrations (0, 6.25, 12.5, 25, 50, and 100 ng/mL); **d** Quantitative determination of HA/LF@C_6_-NPs uptake by NCM-460 cells after 4 h incubation at different times (0, 0.25, 0.5, 1, 2, and 4 h). **C** Qualitative analysis of uptake by RWPE-1 cells incubated with Free C_6_, LF@C_6_-NPs, and HA/LF@C_6_-NPs with or without LF. **D** Quantitative analysis of uptake by RWPE-1 cells. **a** incubated with Control, Free C_6_, LF@C_6_-NPs, and HA/LF@C_6_-NPs; **b** incubated with LF@C_6_-NPs and HA/LF@C_6_-NPs with or without LF; **c** Quantitative determination of HA/LF@C_6_-NPs uptake by RWPE-1 cells after 4 h incubation at different concentrations (0, 6.25, 12.5, 25, 50, and 100 ng/mL); **d** Quantitative determination of HA/LF@C_6_-NPs uptake by RWPE-1 cells after 4 h incubation at different times (0, 0.25, 0.5, 1, 2, and 4 h). Data are represented as the mean ± SEM (n = 3). *p < 0.05; **p < 0.01

The qualitative results of CLMS can be seen in Fig. 2A. The green fluorescence of the free C_6_ group in NCM-460 cells was weaker than that of the LF@C_6_-NPs and HA/LF@C_6_-NPs groups, with the highest fluorescence intensity in the LF@C_6_-NPs group, indicating that the uptake of LF@C_6_-NPs was strongest in NCM-460 cells. This may be because the HA and LF of the HA/LF@C_6_-NPs group bind and occupy part of the LF sites through electrostatic interactions, making the fluorescence intensity of the HA/LF@C_6_-NPs group weaker than that of the LF@C_6_-NPs group. To demonstrate whether the uptake efficiency was enhanced due to the presence of LFR, we pretreated NCM-460 cells with free LF to bind to the LFR on the cells and observed a decrease in the fluorescence intensity of the LF@C_6_-NPs and HA/LF@C_6_-NPs groups compared with the untreated group, indicating that the LFR of NCM-460 cells increased the uptake efficiency of LF@C_6_-NPs.

The qualitative results of FCM are shown in Fig. 2B. The green fluorescence was much higher in the LF@C_6_-NPs and HA/LF@C_6_-NPs groups than in the free C_6_ group (*p* < 0.01), which is similar to the results of CLSM. The uptake efficiency of LF@C_6_-NPs and HA/LF@C_6_-NPs was significantly lower in NCM-460 cells pretreated with LF compared to the untreated group (*p* < 0.01), which is identical to the results of CLSM, showing the effective uptake of LF@C_6_-NPs by NCM-460 cells because of the surface LFR. Furthermore, the fluorescence intensity of the HA/LF@C_6_-NPs group was enhanced with increasing C_6_ concentration (from 6.25 to 100 ng/mL) and time (from 0 ng/mL to 4 h), confirming concentration- and time-dependent uptake by NCM-460 cells. The above results suggest that HA/LF@C_6_-NPs can pass smoothly through the rectum and be absorbed by intestinal epithelial cells, which helps to identify the target prostate.

The uptake of RWPE-1 cells resembled that of NCM-460 cells. The CLMS results (Fig. 2C) showed that compared to the free C_6_ group, RWPE-1 cells showed the highest uptake efficiency of LF@C_6_-NPs, followed by HA/LF@C_6_-NPs. The fluorescence intensity of the above two groups was reduced in RWPE-1 cells preincubated with LF, indicating that the LFR of RWPE-1 cells improved the uptake of LF@C_6_-NPs. The FCM results (Fig. 2D) also showed high LF@C_6_-NPs and HA/LF@C_6_-NPs uptake by RWPE-1 cells compared with the free C_6_ group (*p* < 0.01); the uptake of HA/LF@C_6_-NPs by RWPE-1 cells was also concentration- and time- dependent. In conclusion, the process of intestinal absorption of HA/LF@C_6_-NPs to the prostate gland at the lesion site was achieved.

#### Uptake of HA/LF@EMO-NPs by CD44 receptor-positive RAW 264.7 cells

**Fig. 3:**
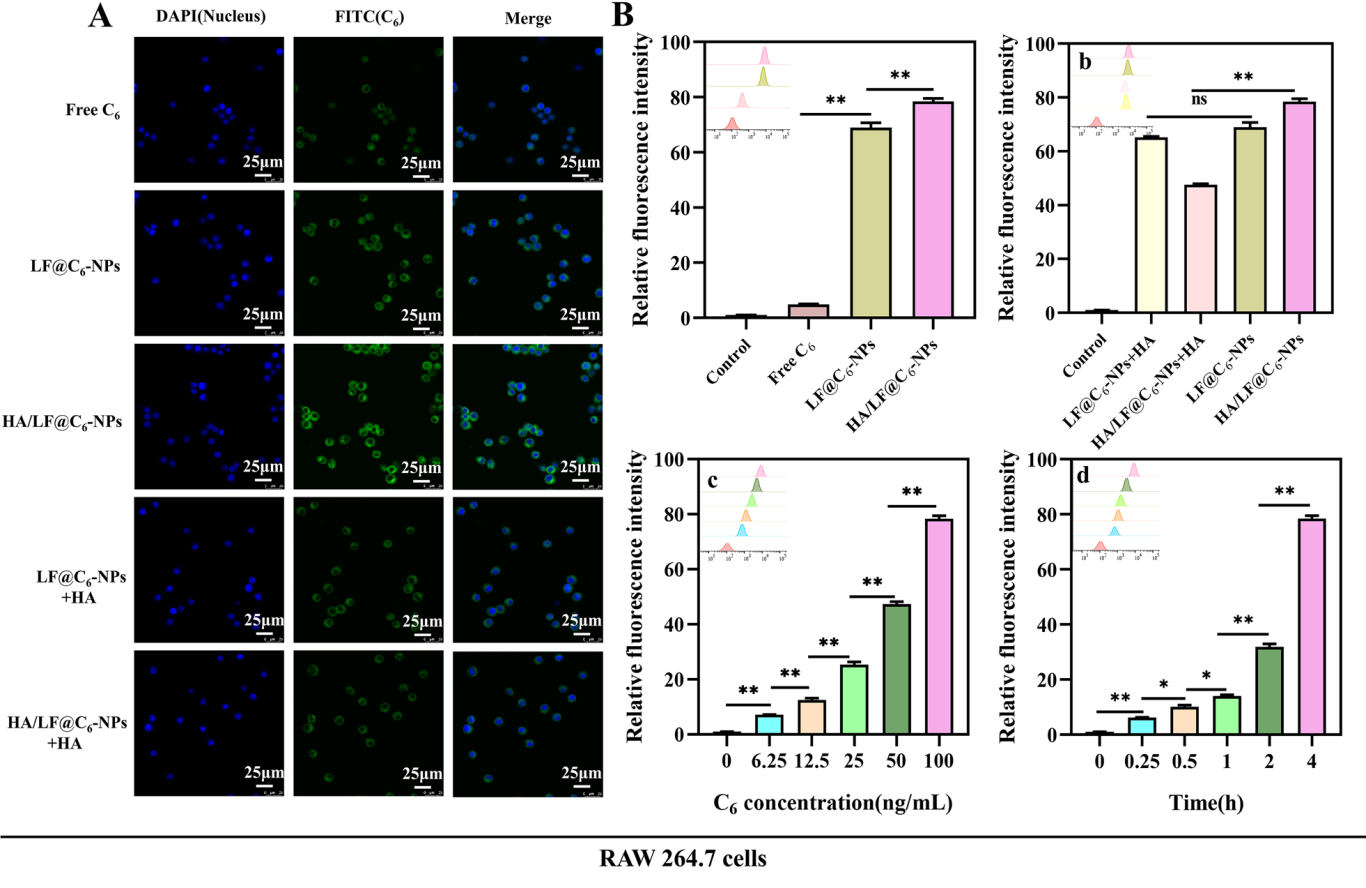
Cellular uptake evaluation of RAW 264.7 cells. **A** Qualitative analysis of uptake by RAW 264.7 cells incubated with Free C_6_, LF@C_6_-NPs, or HA/LF@C_6_-NPs with or without HA. **B** Quantitative analysis of uptake by RAW 264.7 cells. **a** incubated with Control, Free C_6_, LF@C_6_-NPs, and HA/LF@C_6_-NPs; **b** incubated with LF@C_6_-NPs and HA/LF@C_6_-NPs with or without HA; **c** Quantitative determination of HA/LF@C_6_-NPs uptake by RAW 264.7 cells after 4 h incubation at different concentrations (0, 6.25, 12.5, 25, 50, and 100 ng/mL); **d** Quantitative determination of HA/LF@C_6_-NPs uptake by RAW 264.7 cells after 4 h incubation at different times (0, 0.25, 0.5, 1, 2, and 4 h). Data are represented as the mean ± SEM (n = 3). *p < 0.05; **p < 0.01

CD44 receptors are widely present in inflammatory cells and are able to bind specifically to their major ligand HA [33]. In CNP, cells with inflammatory infiltrates have high CD44 receptor expression, but epithelial cells rarely express it [31]. In this study, we investigated the in vitro cellular uptake efficiency of HA/LF@EMO-NPs using RAW 264.7 cells. In addition, to demonstrate the targeting ability of HA for HA/LF@EMO-NPs, we preincubated HA with RAW 264.7 cells and observed the fluorescence intensity changes. After incubation of RAW 264.7 cells with free C_6_, LF@C_6_-NPs and HA/LF@C_6_-NPs (C_6_ 100 ng/mL) for 4 h, we detected stronger fluorescence intensity of LF@C_6_-NPs and HA/LF@C_6_-NPs than free C_6_ using CLMS (Fig. 3A), with the highest fluorescence of HA/LF@C_6_-NPs. The excellent absorption efficiency of HA/LF@C_6_-NPs employing RAW 264.7 cells was confirmed by the reduced fluorescence intensity of the HA preincubation group compared to the untreated group. The FCM results (Fig. 3B) remained consistent with the CLMS results. Moreover, we observed the same concentration- and time-dependent uptake of HA/LF@C_6_-NPs in RAW 264.7 cells. These results suggest that HA surface functionalization can improve the cellular uptake efficiency of HA/LF@EMO-NPs in RAW 264.7 cells, facilitating medication entry into the inflamed prostate tissue and locating the therapeutic target location.

In conclusion, our study meticulously replicated the transrectal absorption process of the formulated drug into the prostate while precisely targeting inflammatory cells using three distinct cell lines. This approach showcases a comprehensive strategy encompassing multiple levels to successfully achieve the delivery of medication to the intended destination.

### In vitro NPs transport

**Fig. 4:**
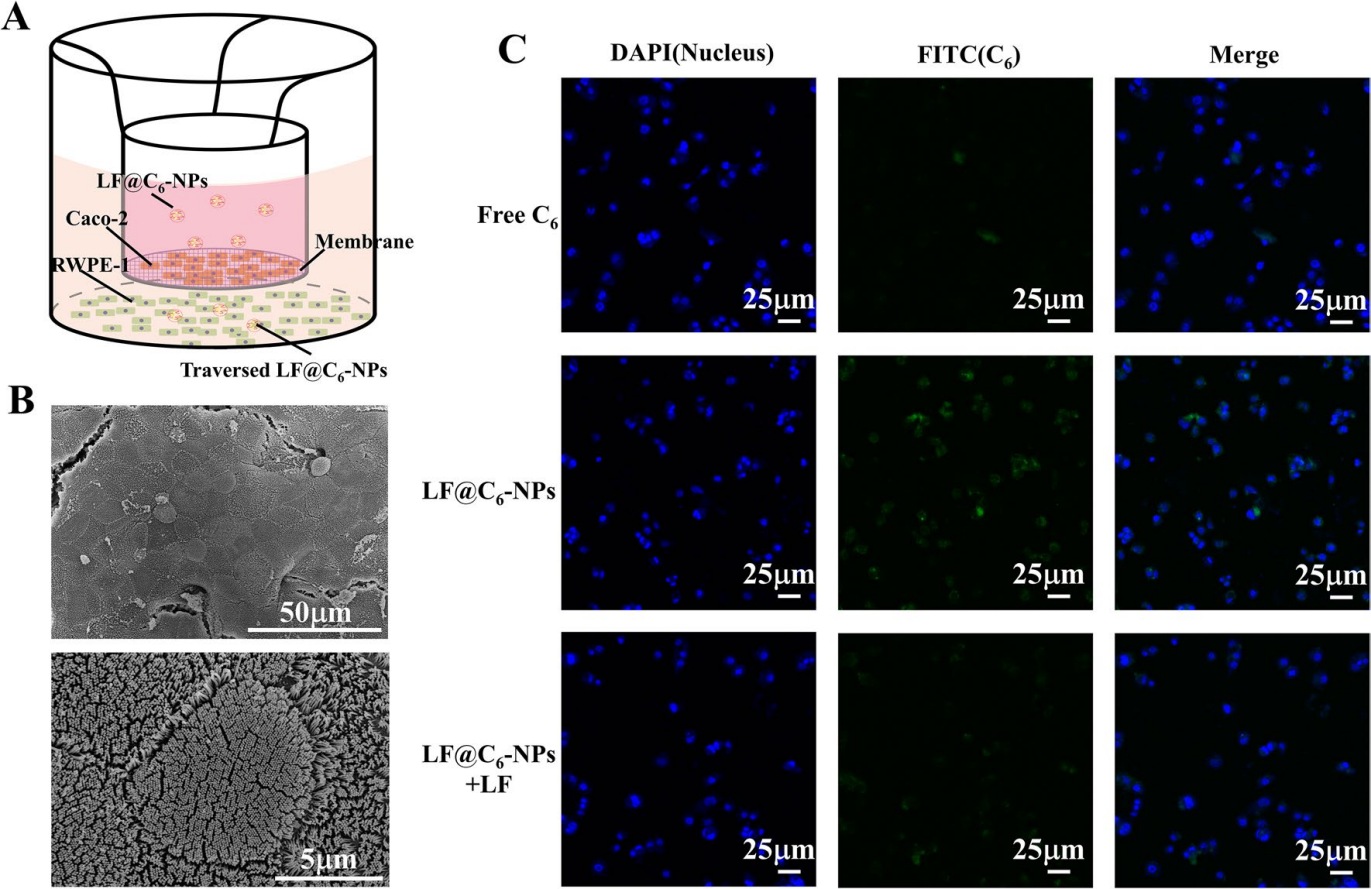
NPs successfully traverses the intestinal epithelial cells and accumulates in the prostate tissue. **A** Illustration of in vitro the NPs transfer. **B** SEM of the in vitro intestinal epithelial cell monolayer model. **C** Qualitative analysis of uptake by RWPE-1 cells incubated with Free C_6_, LF@C_6_-NPs with or without LF

In this study, we mainly relied on LF binding to LFR on intestinal epithelial cells and prostate epithelial cells for effective delivery of NPs. In order to prove that NPs escaped from intestinal epithelial cells and reached prostate tissues, we verified it by establishing an in vitro model. The in vitro NPs transfer process is shown in Fig. 4A. SEM results (Fig. 4B) showed that we successfully established the intestinal epithelial cell monolayer model. CLSM results (Fig. 4C) showed that the fluorescence intensity was strongest in the LF@C_6_-NPs group in the lower chamber, and weakened in the LF-saturated group, suggesting that the drug was able to be transported to the prostate epithelium after uptake by the intestinal epithelial cells.

### In vitro anti-inflammatory efficacies and mechanisms of NPs

**Fig. 5:**
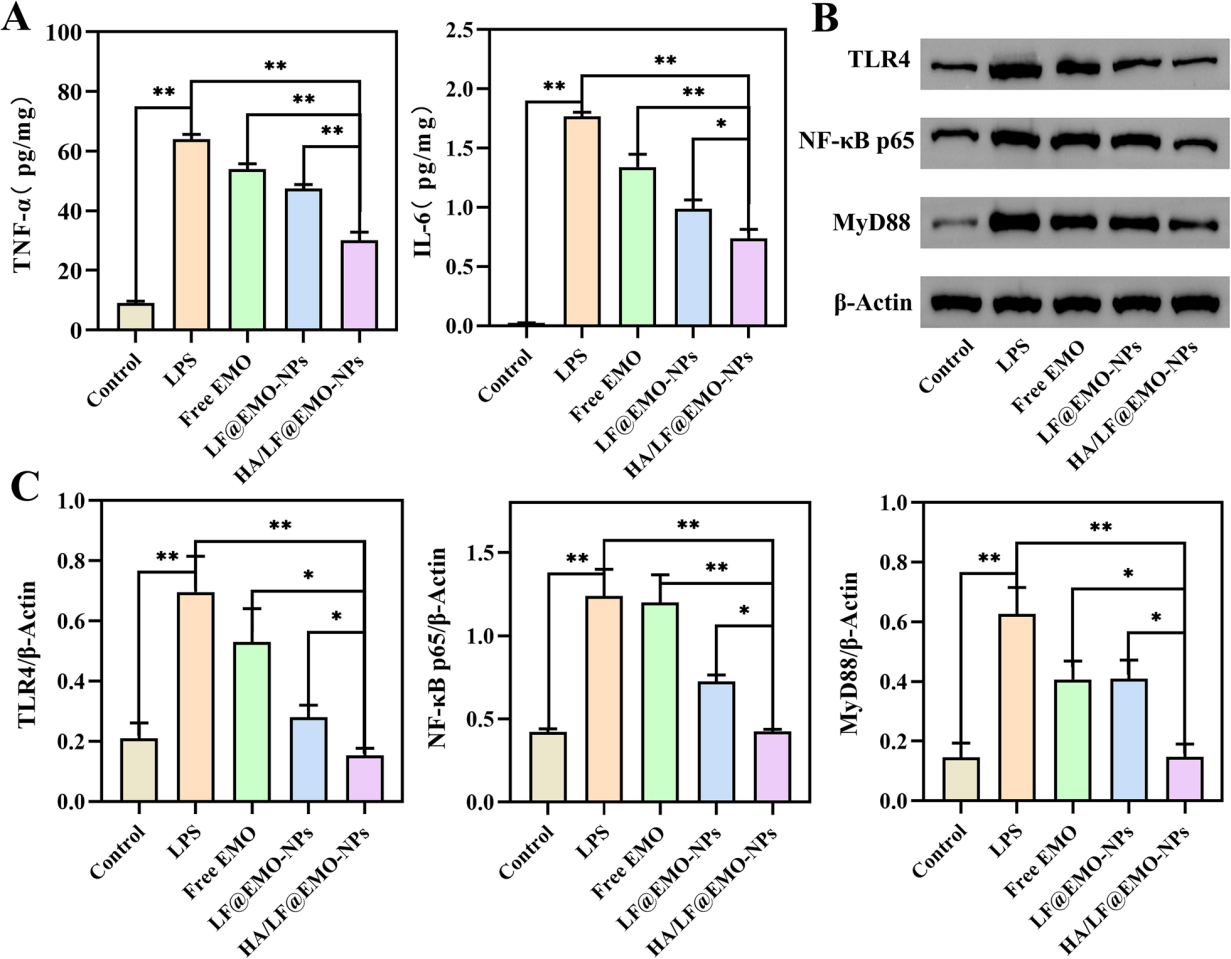
In vitro anti-inflammatory efficacies and mechanisms of NPs**. A** Histogram analysis of changes in inflammatory cytokines (TNF-α, IL-6) in the inflammatory RAW 264.7 cells. **B** Western blotting results. **C** Histogram analysis of the influence of each group on the changes of TLR4, MyD88 and NF-κB p65 protein expression in RAW 264.7 cells. Data are represented as the mean ± SEM (n = 6). *p < 0.05; **p < 0.01

The in vitro pharmacologic research of NPs was conducted by using LPS-induced RAW264.7 cells, The results were shown in Fig. 5A as well as shown as follows, the levels of TNF-α, IL-6 in the LPS group were significantly higher than those in the control group (*p* < 0.01).The expression of TNF-α and IL-6 in the HA/LF@EMO-NPs treatment group was more significantly inhibited than that in the other treatment groups (*p* < 0.01), suggesting that the HA/LF@EMO-NPs can effectively reduce the levels of inflammatory factors, and have a favorable therapeutic effect on the inflammatory RAW 264.7 cells. Western blotting results (Fig. 5B, C) showed that the expression levels of TLR4, Myd88 and NF-κB p65 proteins were significantly higher in the model group than in the normal group (*p* < 0.01). In addition, the expression levels of these three proteins were reduced after drug treatment, in which the HA/LF@EMO-NPs group had the best effect in inhibiting protein expression. The above results suggest that the inhibitory effect of HA/LF@EMO-NPs on LPS-induced inflammatory RAW 264.7 cells may be produced by inhibiting these proteins associated with the TLR4/NF-κB signaling pathway.

### Targeting ability of gel in inflamed prostate tissues

**Fig. 6:**
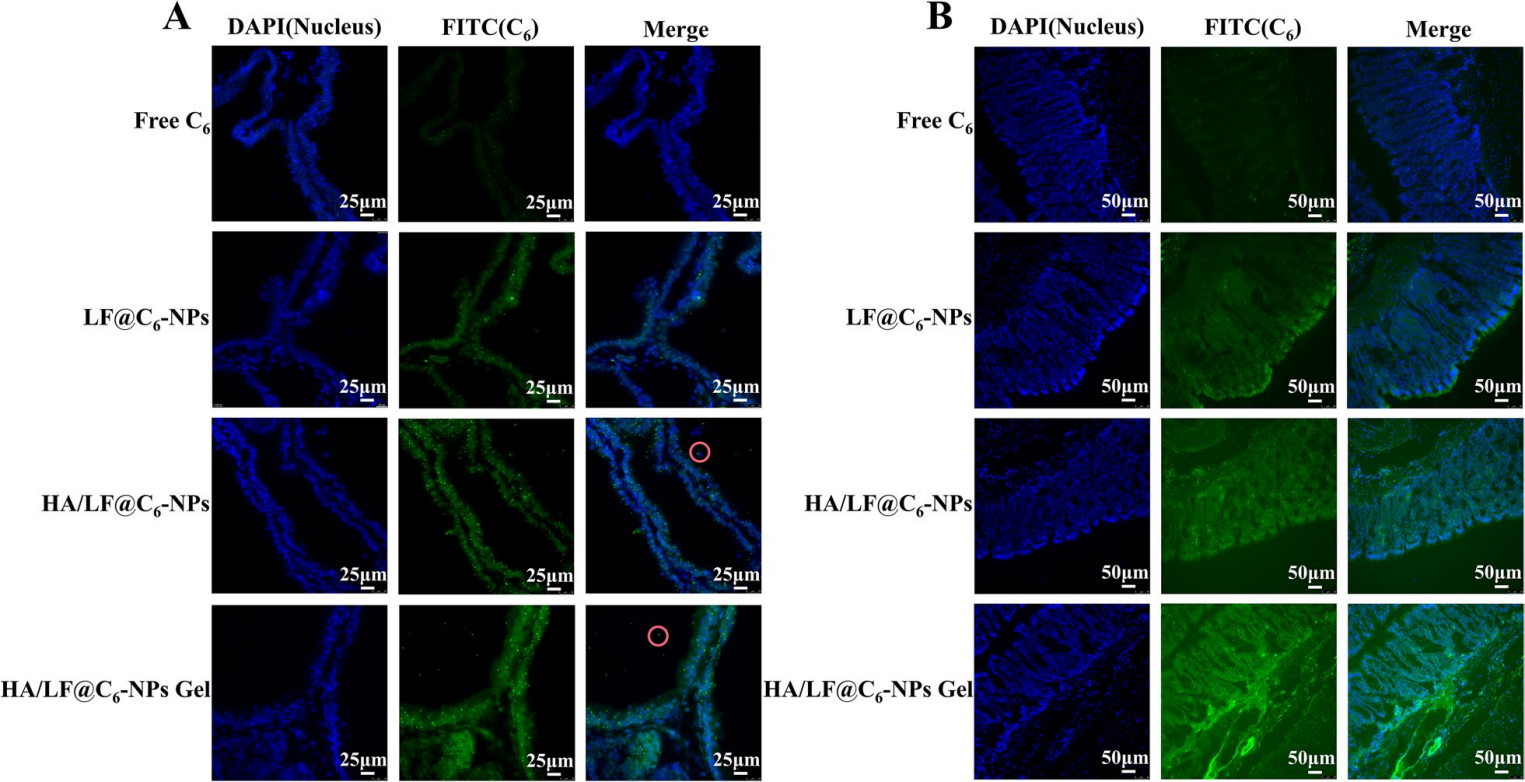
Evaluation of biodistribution in vivo. **A** Frozen sections of prostate tissues after drug administration. **B** Frozen sections of rectal tissues after drug administration. Green, C_6_; blue, DAPI (Nucleus). (n = 6)

Rectal delivery of medications for CNP depends on the efficient retention or targeting of therapeutic medicines in the prostate and rectal locations. We used nano-formulations with fluorescent C_6_ instead of nonfluorescent EMO and observed the results of frozen sections after treatment with different C_6_ preparations by CLMS to assess whether the HA/LF@C_6_-NPs Gel was retained in the prostate and rectal tissues. The green fluorescence signal of prostate and rectal tissues was significantly stronger in the HA/LF@C_6_-NPs and HA/LF@C_6_-NPs Gel groups than in the free C_6_ and LF@C_6_-NPs groups after 24 h of administration to rats (Fig. 6A, B). Interestingly, some fluorescence was also found inside the prostate lumen in the HA/LF@C_6_-NPs and HA/LF@C_6_-NPs Gel groups, indicating that the two groups could effectively deliver the drug to the inside of the prostate lumen. In addition, the green fluorescence intensity of prostate and rectal tissues in the Gel group was slightly stronger than that in the NPs group, indicating that rectal thermosensitive hydrogel administration could increase the drug retention effect. In addition, after 24 h of drug administration to rats, the green fluorescence signals in the heart, liver, spleen, lung and kidney tissues were weaker in all formulation groups, which were much lower than the fluorescence intensity in the prostate and rectal tissues (as showed in Additional file 1: Fig. S3).

Furthermore, we have examined the content of EMO in the residual gel by HPLC after different timepoints (3, 6, 12, 24 h) of rectal administration of HA/LF@EMO-NPs Gel. The results (as showed in Additional file 1: Fig. S4) showed that EMO is still present at the rectal site after 24 h, suggesting that the formulation is able to be retained in the rectum for at least 24 h. Therefore, it is evident that by daily rectal administration of HA/LF@EMO-NPs Gel, EMO can be effectively accumulated in the rectum of CNP rats. In conclusion, the nanogel rectal drug delivery system designed in this study could effectively retain in the rectum and promote the absorption of the drug in the rectal site, as well as have a good prostate-targeting effect.

### In vivo therapeutic evaluation

**Fig. 7:**
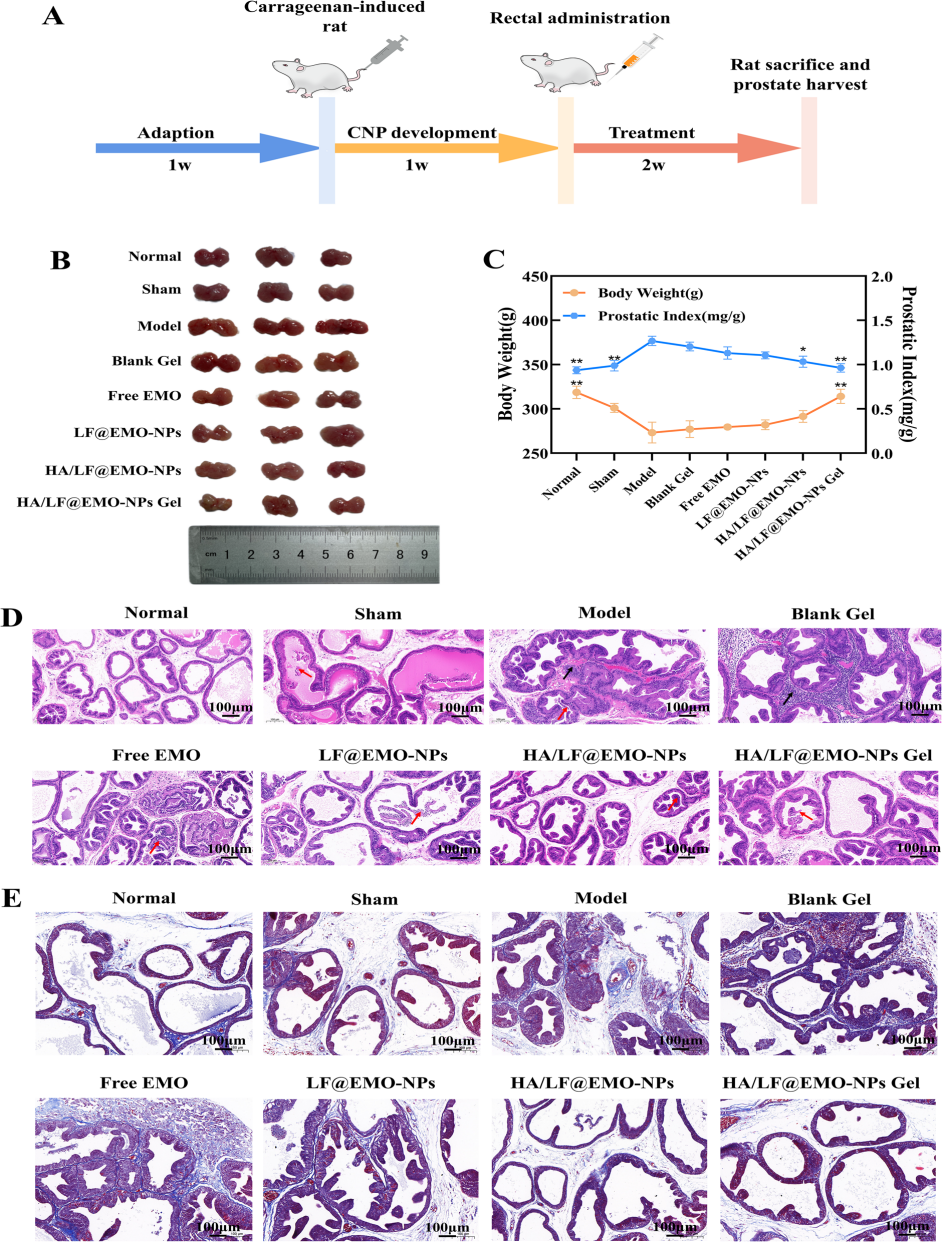
In vivo therapeutic results of HA/LF@EMO-NPs Gel in CNP treatment. **A** Experimental design of drug therapy against CNP. **B** Photographs of prostates after rectal administration with different formulations. **C** Curve chart analysis of bodyweight and PI scores among the eight formulations. **D** H&E staining of prostatic sections among the eight formulations. black arrows: inflammatory cell infiltration; red arrow: shedding of glandular epithelial cells. **E** Masson staining of prostatic sections among the eight formulations. Data are represented as the mean ± SEM (n = 6). *p < 0.05; **p < 0.01

A traditional model for CNP is the delivery of the chemical inflammatory agent carrageenan to the rat prostate [47, 48]. Therefore, this study used the carrageenan-induced CNP model in rats to evaluate the curative effects of HA/LF@EMO-NPs Gel in vivo. We demonstrated the procedure of animal modeling and medication administration (Fig. 7A). The prostate was removed at the final point, as shown in Fig. 7B, and it can be seen that the prostate was the largest in the model group. Compared with the model group, the rats in the HA/LF@EMO-NPs Gel group had smaller prostates, similar to the normal and sham groups. On the last day of rectal administration, the changes in body weight and PI score of rats in each group are shown in Fig. 7C. Compared with the model group, rats in the HA/LF@EMO-NPs Gel group had significantly higher body weight (*p* < 0.01) and significantly lower PI score (*p* < 0.01), both of which were more similar to the normal and sham groups. Taken together, these results indicate that the HA/LF@EMO-NPs Gel treatment exerted a positive and beneficial effect on carrageenan-induced CNP rats.

Furthermore, we employed H&E and Masson staining techniques to examine the pathological alterations occurring within the prostate gland across the eight rat groups. The H&E staining outcomes (Fig. 7D) provided valuable insights: the prostate glands of rats in the normal group exhibited an intact structural framework devoid of evident inflammatory changes. In contrast, the model and blank Gel groups displayed pronounced infiltration of inflammatory cells within the interstitial spaces of the prostate, accompanied by edema, and detachment and necrosis of the glandular epithelium; compared with the model and blank Gel groups, the HA/LF@EMO-NPs Gel treatment group showed enhanced structural integrity of the prostate gland and reduced inflammatory cell infiltration. In addition, the HA/LF@EMO-NPs Gel group showed better remission of CNPs than the other treatment groups. Quantitative analysis (Additional file 1: Fig. S5A) of the score of prostate tissues showed statistically significant differences between the model group compared with the normal and sham groups (*p* < 0.01). Compared to the HA/LF@EMO-NPs Gel group, the remaining intervention groups also showed statistically significant differences (*p < 0.05; **p < 0.01). Masson staining (Fig. 7E) of rats in the model and blank Gel groups showed more collagen fibers stained blue in the interstitial region of the prostate, while relatively fewer in the HA/LF@EMO-NPs and HA/LF@EMO-NPs Gel groups, similar to the normal and sham groups, indicating that the EMO-loaded preparation exerted an antifibrotic effect to some extent in the treatment of CNP. The results (Additional file 1: Fig. S5B) of collagen fiber area percentage also suggested that EMO-loaded preparations exerted some antifibrotic effect in CNP treatment (*p < 0.05; **p < 0.01). To ensure the safety of the preparation, we scrutinized sections from the major organs (heart, liver, spleen, lung, and kidney) of CNP rats following treatment. Notably, no significant pathological damage was detected in any of these organs (Additional file 1: Fig. S6), underscoring the safety profile of the administered treatment.

### Gel exerts anti-inflammatory effects by inhibiting the TLR4-linked NF-κB signaling pathway

**Fig. 8:**
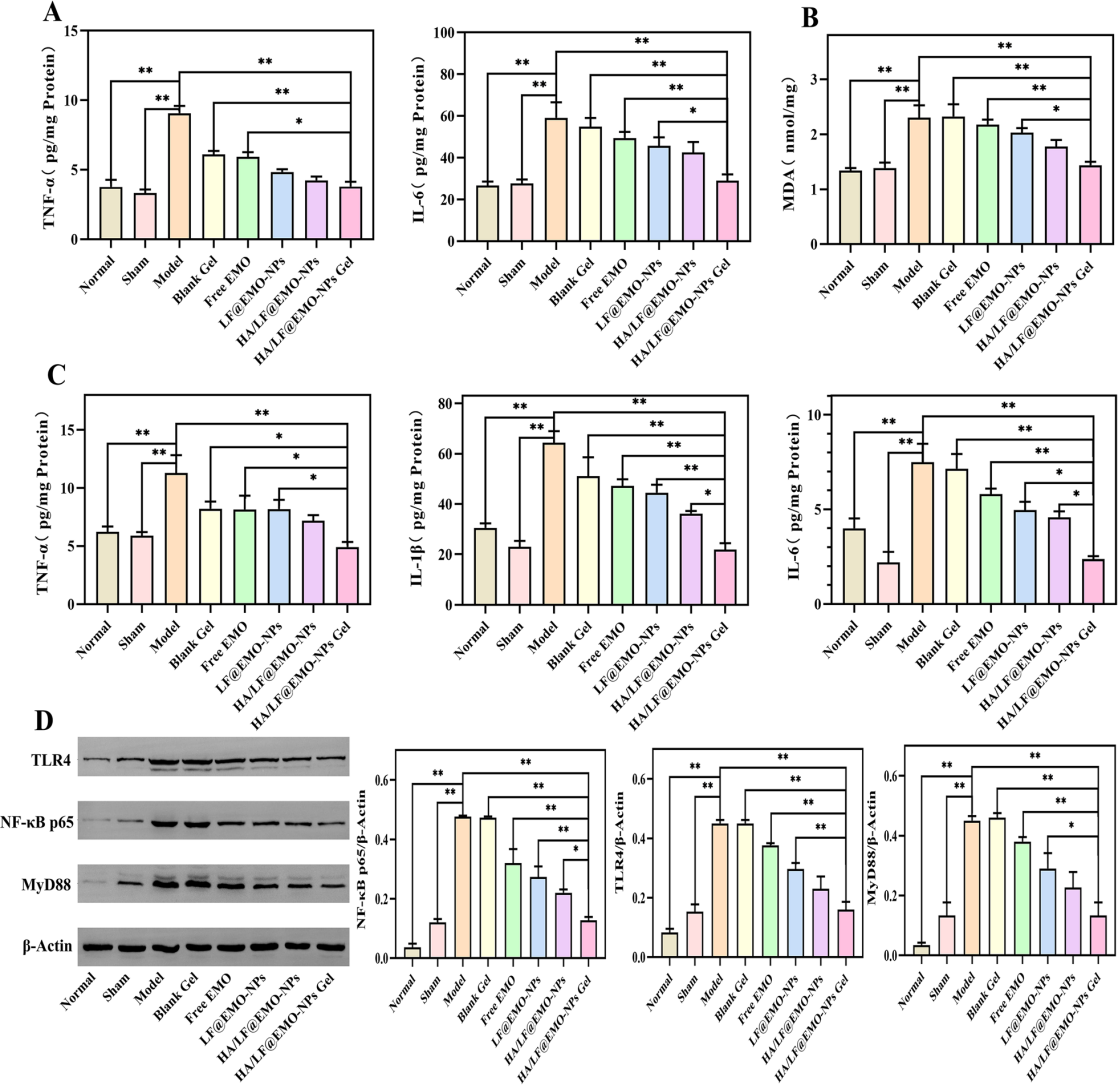
Inflammatory cytokine changes, oxidative indicator changes, and protein expression results in the prostate after HA/LF@EMO-NPs Gel treatment for CNP. **A** Histogram analysis of changes in inflammatory cytokines (TNF-α and IL-6) in the rat serum between the eight formulations. **B** Histogram analysis of changes in oxidative indicator (MDA) in the rat prostate between the eight formulations. **C** Histogram analysis of changes in inflammatory cytokines (TNF-α, IL-1β, and IL-6) in the rat prostate between the eight formulations. **D** Western blot results and histogram analysis of rat prostatic protein expression (MyD88, TLR4, NF-κB p65, and β-Actin) across the eight formulations. Data are mean ± SEM (n = 6). *p < 0.05; **p < 0.01

The TLR4/NF-κB signaling pathway has a key role in oxidative stress and the inflammatory response and can suppress the inflammatory response by regulating the TLR4/NF-κB signaling pathway [15]. It also plays a significant role in the pathogenesis of CNP [49, 50]. As shown in Fig. 8A, the TNF-α and IL-6 levels of serum in the model group were significantly higher than those in the normal and sham groups (*p < 0.05; **p < 0.01). The inhibitory effects of TNF-α and IL-6 were stronger in the HA/LF@EMO-NPs Gel treated group than in the other treatment groups, suggesting that the HA/LF@EMO-NPs Gel could effectively reduce the serum levels of inflammatory factors in CNP rats. Figure 8B showed elevated MDA levels of prostate tissues in the model group compared to the normal and sham groups (*p* < 0.01). Remarkably, the HA/LF@EMO-NP Gel exhibited superior inhibition of MDA, underscoring the antioxidant efficacy of EMO in CNP rats. Additionally, as shown in Fig. 8C, the TNF-α, IL-1β, and IL-6 levels of prostate tissues in the model group were considerably greater than those in the normal and sham groups (*p* < 0.01). The HA/LF@EMO-NPs Gel treatment group had a more pronounced expression of TNF-α, IL-1β, and IL-6 than the rest of the treatment groups (*p < 0.05; **p < 0.01), indicating that HA/LF@EMO-NPs Gel could effectively transport EMO to the site of prostate inflammation and reduce the level of inflammatory factors in CNP rats and had the best therapeutic effect on CNP.

We analyzed typical proteins (TLR4, MyD88 and NF-κB) to investigate the effect of HA/LF@EMO-NPs Gel on the TLR4/NF-κB signaling pathway using Western blotting (Fig. 8D). The model group exhibited significantly elevated expression of TLR4, MyD88, and NF-κB p65 compared to the normal and sham groups (*p* < 0.01). The expression of NF-κB p65 was significantly inhibited in the HA/LF@EMO-NPs Gel group compared with the other treatment groups (*p < 0.05; **p < 0.01). Except for the HA/LF@EMO-NPs treatment group, the HA/LF@EMO-NPs Gel treatment group significantly inhibited the expression of TLR4 and MyD88 compared to the other groups (*p < 0.05; **p < 0.01). In summary, HA/LF@EMO-NP Gel emerged as the most efficacious approach for addressing carrageenan-induced CNP in rats, with its anti-inflammatory effects potentially attributed to TLR4/NF-κB signaling pathway inhibition.

## Conclusions

In this study, we developed a thermosensitive rectal hydrogel delivery system with triple-targeting layer NPs for the treatment of carrageenan-induced CNP. Overcoming the current limitations in CNP treatment, particularly the presence of barriers such as the blood-prostate barrier (BPB), required innovative nanodrug delivery techniques to achieve optimal drug administration. Additionally, in contrast to prior intravenous medication administration, we creatively created a thermosensitive rectal hydrogel formulation with LF, Gel, and HA, all of which have positive effects on the treatment of CNP, as listed below. On the one hand, LF may target intestinal epithelial cells, allowing the medicine to be absorbed by the gut and travel to the prostate; LF can also target prostate epithelial cells to deliver the drug to the interior of the prostatic lumen through phagocytosis. The Gel facilitates rectal drug delivery and has strong adhesion, which achieves effective retention and sustained slow release of the drug and helps the uptake of the drug by intestinal epithelial cells. HA, known for its specificity toward inflammatory macrophages, targets these cells within the prostate. This targeted approach offers potent anti-inflammatory effects and complements the overall therapy. Based on these contributions, we conducted both in vivo and in vitro studies, and all three materials were confirmed to achieve effective treatment of CNP through tacit cooperation, with excellent feedback from the uptake of NCM-460, RWPE-1 and RAW 246.7 cells in vitro and the NF-κB signaling pathway in vivo. Therefore, through the combination of LF, Gel, and HA, a good drug delivery concept was achieved, from the effective retention of the drug in the rectum to promote uptake by the intestinal epithelial cells, thus delivering to the prostate; then, through uptake by the prostate epithelial cells into the interior of the prostate, and finally identifying the target macrophages to exert anti-inflammatory effects, perfectly completing the process.

Furthermore, this research capitalized on the recognized anti-inflammatory potential of EMO, which had demonstrated efficacy across a broad spectrum of conditions. This study marks the first to underscore EMO's benefits in CNP treatment and to delve into its capacity to intervene in NF-κB-related pathways for CNP treatment. The development of this innovative drug delivery strategy was facilitated by the availability of a robust rectal drug delivery platform.

### Supplementary Information

**Additional file 1: Fig. S1** A homemade heavy load device for measuring the mechanical strength of Gel. **Fig. S2** Characterizations of the dialysis solution of the Gel. (A) Size of the upper layer of the clear liquid of the Gel. (B) TEM of the upper layer of the clear liquid of the Gel. **Fig. S3** Frozen sections of heart, liver, spleen, lung, kidney tissues after drug administration. Green, C_6_; blue, DAPI (Nucleus). (n = 6). **Fig. S4** Ratio of EMO/Gel in rectum of CNP rats at different time points. Data are represented as the mean ± SD (n = 6). *p < 0.05; **p < 0.01. **Fig. S5** Pathological scoring. (A)The score of prostatic inflammations among the eight formulations. (B) Percentage of area (%) of collagen fibers among the eight formulations. Data are represented as the mean ± SEM (n = 6). *p < 0.05; **p < 0.01. **Fig. S6** H&E staining of the heart, liver, spleen, lung, and kidney from various groups.

## Data Availability

All data generated and analyzed during this research are included in this published article and its additional file.
