# Correction: Thermosensitive hydrogel with emodin-loaded triple-targeted nanoparticles for a rectal drug delivery system in the treatment of chronic non-bacterial prostatitis

**DOI:** 10.1186/s12951-024-02484-7

**Published:** 2024-05-17

**Authors:** Yan Ye, Wenzhen Zhong, Ruifeng Luo, Hongzhi Wen, Ziyang Ma, Shanshan Qi, Xiaoqin Han, Wenbiao Nie, Degui Chang, Runchun Xu, Naijing Ye, Fei Gao, Peihai Zhang

**Affiliations:** 1 TCM Regulating Metabolic Diseases Key Laboratory of Sichuan Province, Hospital of Chengdu University of Traditional Chinese Medicine, Chengdu, 610072 China; 2 State Key Laboratory of Southwestern Chinese Medicine Resources, Pharmacy School, Chengdu University of Traditional Chinese Medicine, Chengdu, 611130 China

**Correction: Journal of Nanobiotechnology (2024) 22:33**

10.1186/s12951-023-02282-7

Following publication of the original article, details for affiliation of author Yan Ye was incorrectly given as “ State Key Laboratory of Southwestern Chinese Medicine Resources, Pharmacy School, Chengdu University of Traditional Chinese Medicine, Chengdu, 611,130, China” but should have been “TCM Regulating Metabolic Diseases Key Laboratory of Sichuan Province, Hospital of Chengdu University of Traditional Chinese Medicine, Chengdu 610072, China”.

The original article has been corrected.

