# Supplementary material for: Thermosensitive hydrogel with emodin-loaded triple-targeted nanoparticles for a rectal drug delivery system in the treatment of chronic non-bacterial prostatitis
Source: J Nanobiotechnology. 2024 Jan 18;22:33. doi: 10.1186/s12951-023-02282-7 (PMC10795337; doi:10.1186/s12951-023-02282-7)
Supplement: Supplementary file 1 — Additional file 1: Fig. S1 A homemade heavy load device for measuring the mechanical strength of Gel. Fig. S2 Characterizations of the dialysis solution of the Gel. (A) Size of the upper layer of the clear liquid of the Gel. (B) TEM of the upper layer of the clear liquid of the Gel. Fig. S3 Frozen sections of heart, liver, spleen, lung, kidney tissues after drug administration. Green, C6; blue, DAPI (Nucleus). (n = 6). Fig. S4 Ratio of EMO/Gel in rectum of CNP rats at different time points. Data are represented as the mean ± SD (n = 6). *p < 0.05; **p < 0.01. Fig. S5 Pathological scoring. (A)The score of prostatic inflammations among the eight formulations. (B) Percentage of area (%) of collagen fibers among the eight formulations. Data are represented as the mean ± SEM (n = 6). *p < 0.05; **p < 0.01. Fig. S6 H&E staining of the heart, liver, spleen, lung, and kidney from various groups. [file 12951_2023_2282_MOESM1_ESM.docx]

**Additional Materials**

**Additional Figure 1**


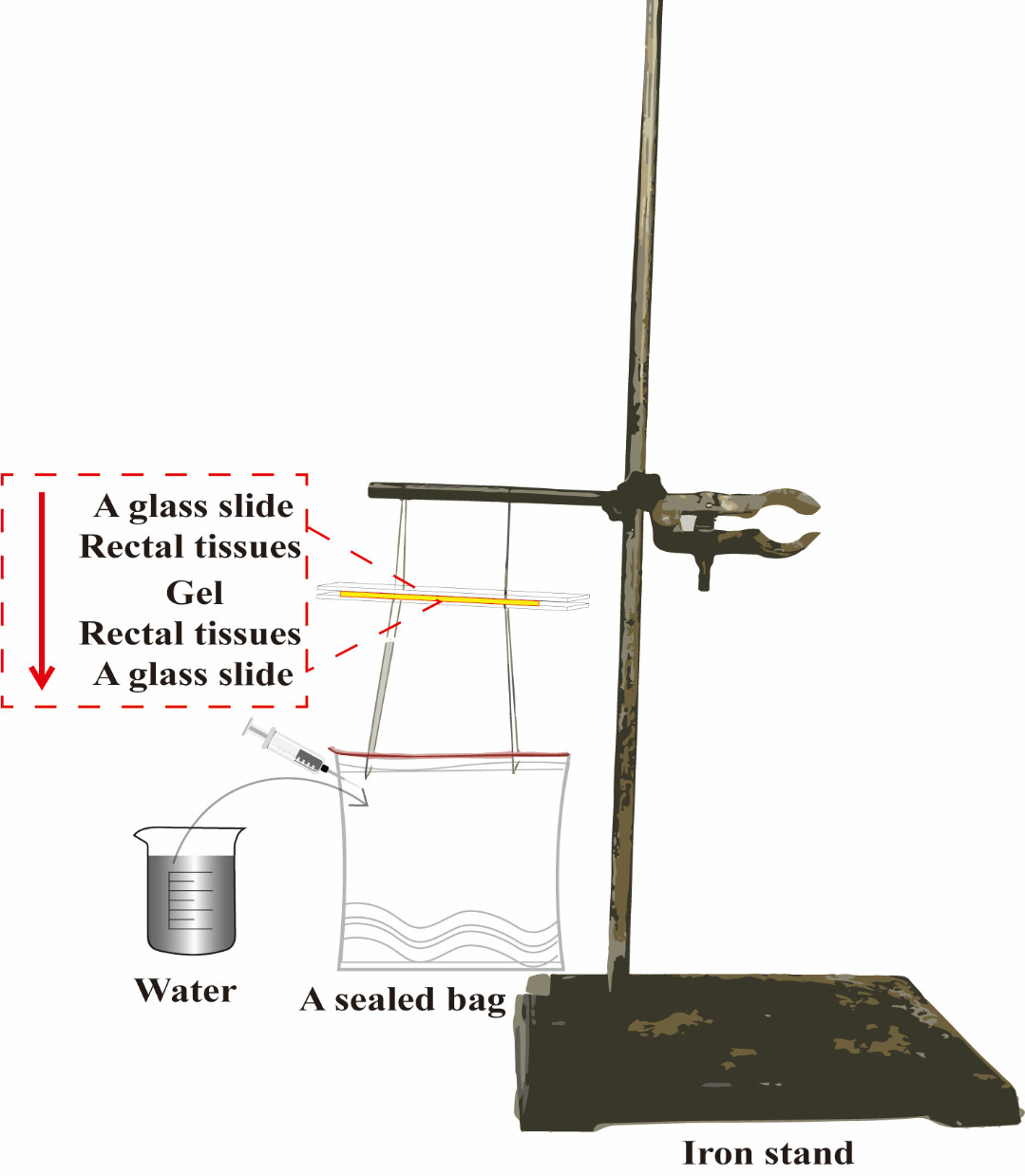


**Fig. S1** A homemade heavy load device for measuring the mechanical strength of Gel.

**Additional Figure 2**

**
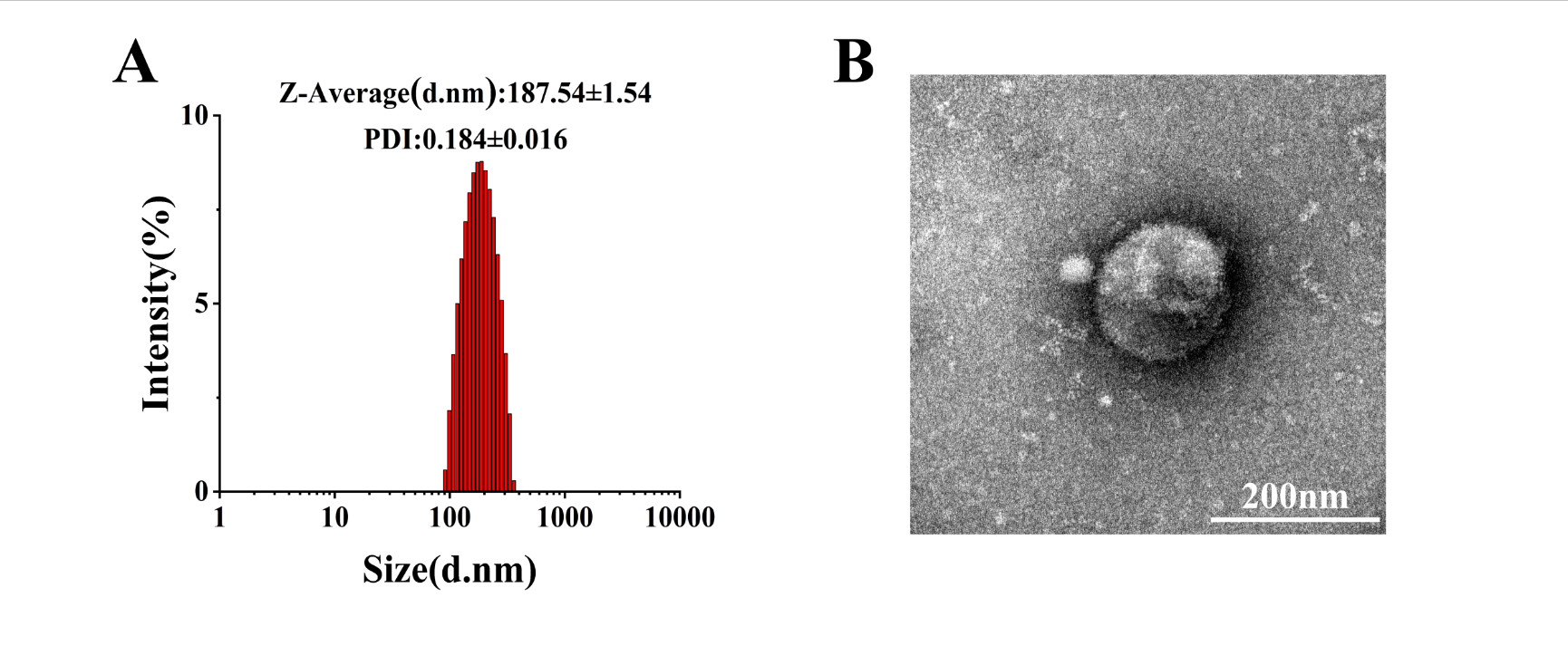
**

**Fig. S2** Characterizations of the dialysis solution of the Gel. **(A)** Size of the upper layer of the clear liquid of the Gel. (B) TEM of the upper layer of the clear liquid of the Gel.

**Additional Figure 3**


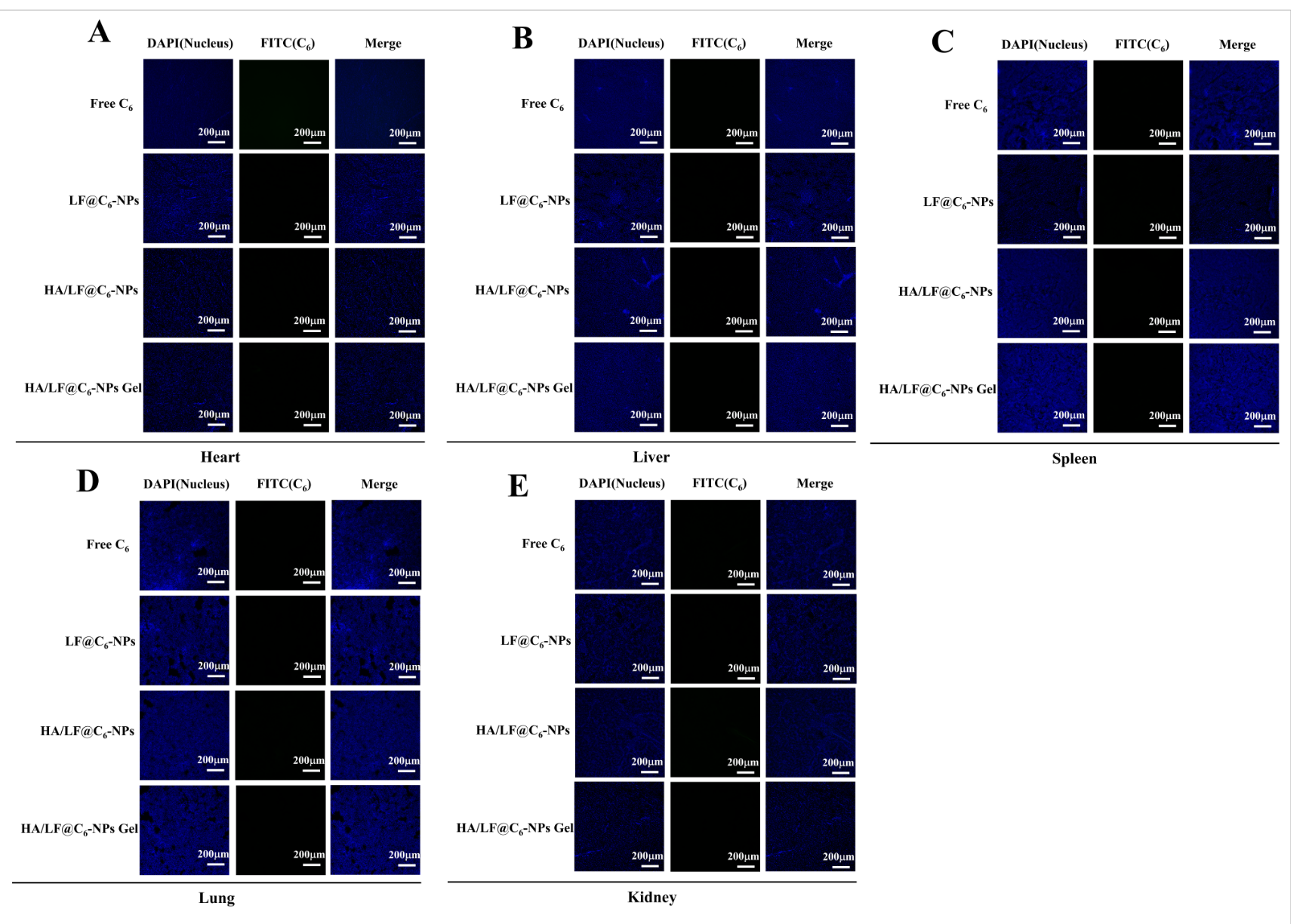


**Fig. S3** Frozen sections of heart, liver, spleen, lung, kidney tissues after drug administration. Green, C_6_; blue, DAPI (Nucleus). (n = 6)

`

**Additional Figure 4**


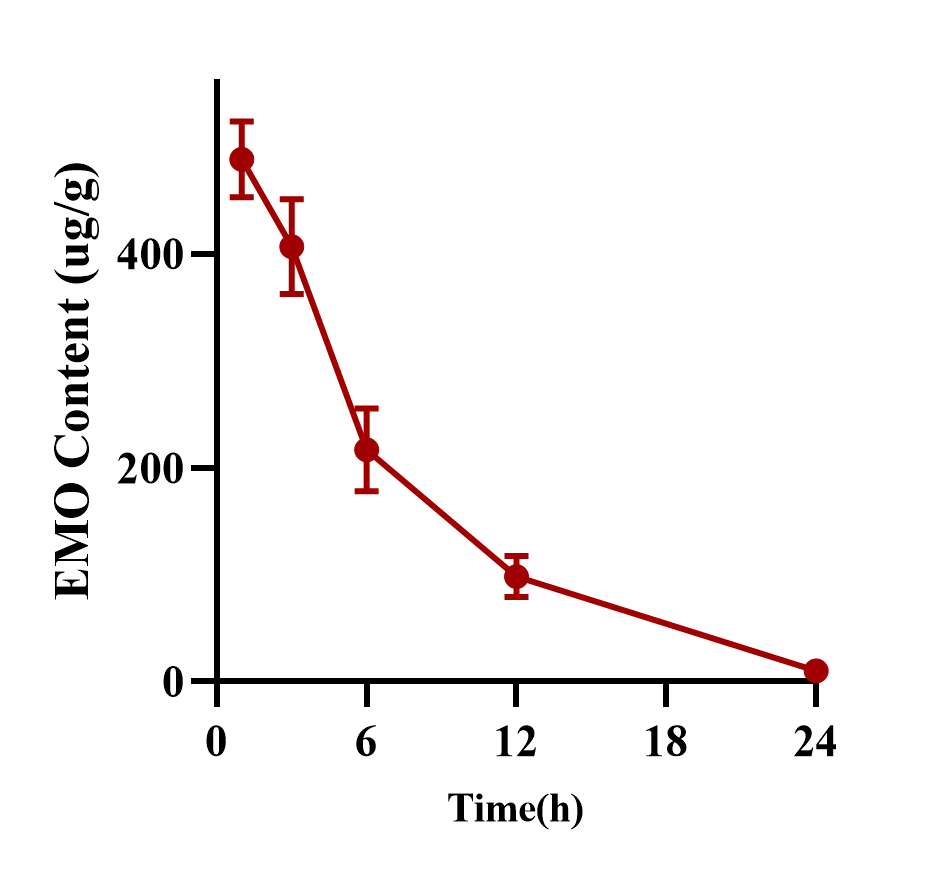


**Fig. S4** Ratio of EMO/Gel in rectum of CNP rats at different time points. Data are represented as the mean ± SD (n = 6). **p* < 0.05; ***p* < 0.01.

**Additional Figure 5**


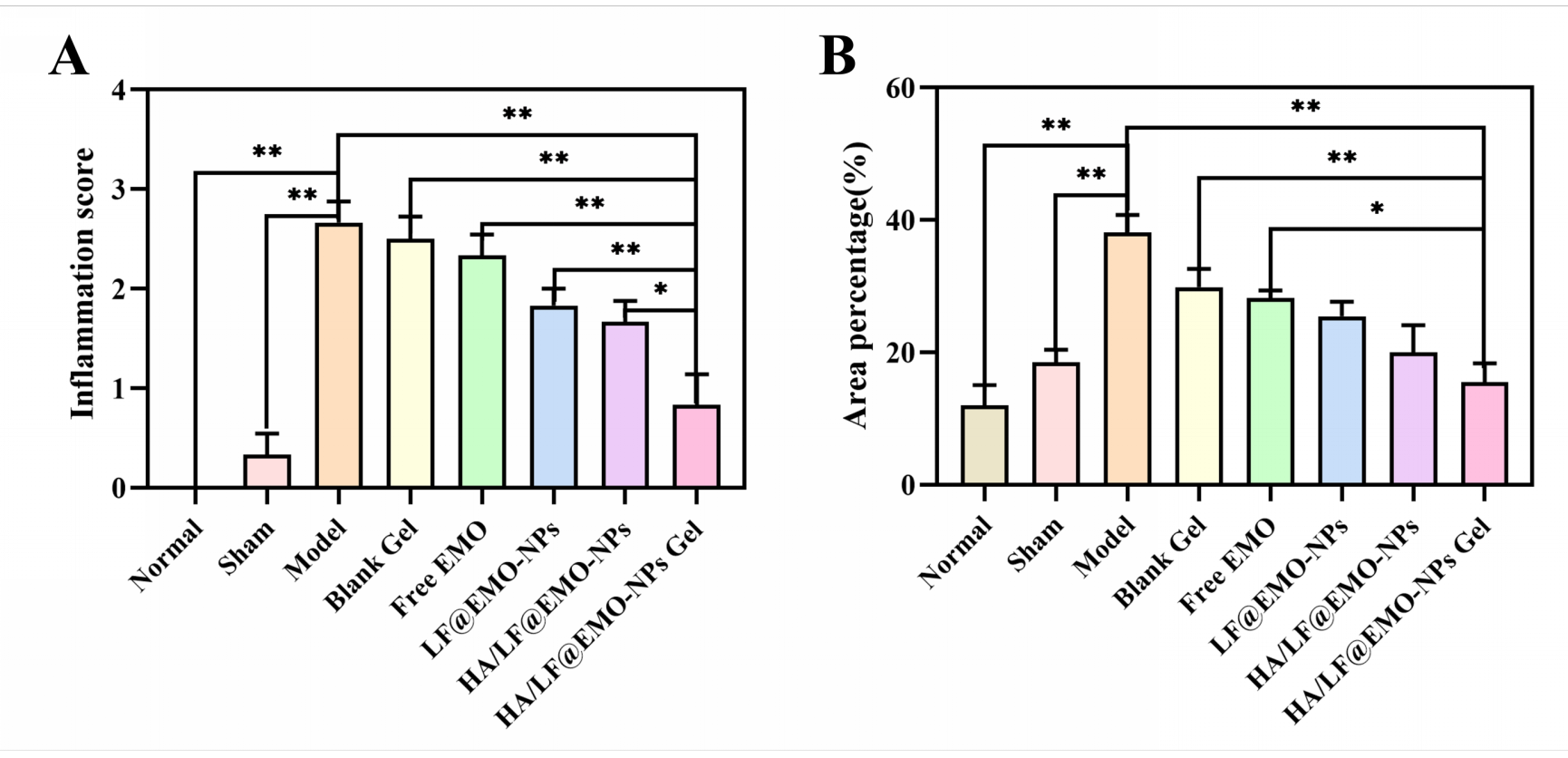


**Fig. S5** Pathological scoring. (A)The score of prostatic inflammations among the eight formulations. (B) Percentage of area (%) of collagen fibers among the eight formulations. Data are represented as the mean ± SEM (n = 6). **p* < 0.05; ***p* < 0.01.

**Additional Figure 6**


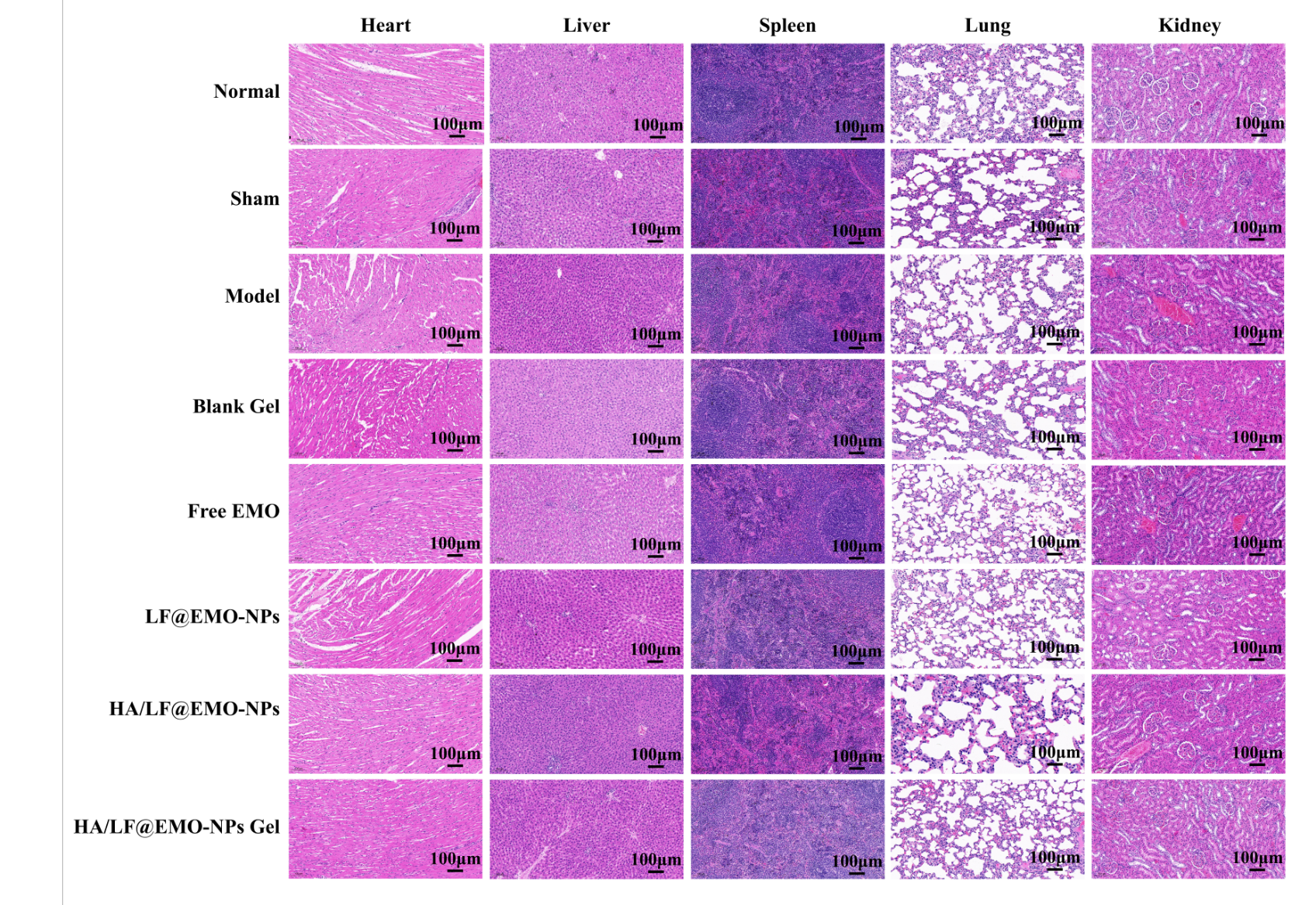


**Fig. S6** H&E staining of the heart, liver, spleen, lung, and kidney from various groups.
